# Nanovaccine administration route is critical to obtain pertinent iNKt cell help for robust anti-tumor T and B cell responses

**DOI:** 10.1080/2162402X.2020.1738813

**Published:** 2020-03-17

**Authors:** Yusuf Dölen, Michael Valente, Oya Tagit, Eliezer Jäger, Eric A. W. Van Dinther, N. Koen van Riessen, Martin Hruby, Uzi Gileadi, Vincenzo Cerundolo, Carl G. Figdor

**Affiliations:** a Department of Tumor Immunology, Radboud Institute for Molecular Life Sciences, Radboud University Medical Center & Oncode Institute, Nijmegen, The Netherlands; b Institute of Macromolecular Chemistry V.v.i., Academy of Sciences of the Czech Republic, Prague 6, Czech Republic; c MRC Human Immunology Unit, Weatherall Institute of Molecular Medicine, University of Oxford, Oxford, UK

**Keywords:** Cancer vaccines, nanoparticle biodistribution, iNKT cells, PD-1, 4-1BB, checkpoint blockade

## Abstract

Nanovaccines, co-delivering antigen and invariant natural killer T (iNKT) cell agonists, proved to be very effective in inducing anti-tumor T cell responses due to their exceptional helper function. However, it is known that iNKT cells are not equally present in all lymphoid organs and nanoparticles do not get evenly distributed to all immune compartments. In this study, we evaluated the effect of the vaccination route on iNKT cell help to T and B cell responses for the first time in an antigen and agonist co-delivery setting. Intravenous administration of PLGA nanoparticles was mainly targeting liver and spleen where iNKT1 cells are abundant and induced the highest serum IFN-y levels, T cell cytotoxicity, and Th-1 type antibody responses. In comparison, after subcutaneous or intranodal injections, nanoparticles mostly drained or remained in regional lymph nodes where iNKT17 cells were abundant. After subcutaneous and intranodal injections, antigen-specific IgG2 c production was hampered and IFN-y production, as well as cytotoxic T cell responses, depended on sporadic systemic drainage. Therapeutic anti-tumor experiments also demonstrated a clear advantage of intravenous injection over intranodal or subcutaneous vaccinations. Moreover, tumor control could be further improved by PD-1 immune checkpoint blockade after intravenous vaccination, but not by intranodal vaccination. Anti PD-1 antibody combination mainly exerts its effect by prolonging the cytotoxicity of T cells. Nanovaccines also demonstrated synergism with anti-4-1BB agonistic antibody treatment in controlling tumor growth. We conclude that nanovaccines containing iNKT cell agonists shall be preferentially administered intravenously, to optimally reach cellular partners for inducing effective anti-tumor immune responses.

## Introduction

iNKT cells comprise a subset of T cells that share both NK cell characteristics and bear an invariant αβ T cell receptor. Instead of protein peptides that are recognized by regular T cells, iNKT cells recognize glycolipids presented by MHC Class I like molecule CD1d. In particular, microorganism-derived glycolipids that contain a sugar α-linked to a lipid tail are amongst the strongest TCR binding antigens.^1^ iNKT cells can be divided into further subsets based on their cytokine secretion profile: iNKT1 cells are the only producers of IFN-y, iNKT2 cells mainly produce IL-4 and iNKT17 cells are the sole producers of IL-17.^2^ iNKT cells have been considered as a potential tool in cancer immunotherapy, making use of their ability to either directly kill tumor cells or to activate NK cells via IFN-y and IL-21 secretion.^3,4^ Furthermore, it has been demonstrated that iNKT cells exert a strong helper function by the secretion of cytokines to generate robust CD8^+^ T cell responses.^5^ Therefore, α-galactosylceramide (α-GalCer) and its analogs thereof have been widely explored as vaccine adjuvants to boost T cell responses.^6^ In this study, we used IMM60 as an iNKT cell agonist as it was shown to have a higher affinity to human iNKT-cell TCR than α-GalCer and results in extended responses both in human and mouse iNKT cells^7^

We and others previously demonstrated that the unique helper properties of iNKT cells could be efficiently exploited by co-encapsulating both antigens and iNKT cell agonists in biodegradable poly(lactic-co-glycolic acid) (PLGA) nanoparticles, thereby greatly enhancing cytotoxic T cell responses independent of CD4^+^ T cell help.^8,9^ Important advantages of such PLGA nanoparticle-based vaccines are: 1) antigens are protected from premature degradation by proteolytic enzymes in the serum, 2) higher amounts of antigen gets delivered to professional antigen-presenting cells, and 3) antigen and adjuvant co-delivery guarantees maturation and activation of DCs thereby providing both TCR and co-stimulatory signals to T cells, resulting in strong anti-tumor responses in preclinical mouse models.^8,10^ On the other hand, particulate vaccines (depending on the size) may not be able to diffuse systemically and different iNKT cell subsets are not equally present in all lymphoid organs thus raising the importance of vaccination site.^11,12^

In most clinical studies DCs or PBMCs were loaded with α-GalCer *ex–vivo* before being administered into patients.^13^ It was reported that intravenously (iv) injected cells led to a higher increase of iNKT cell numbers than intradermally (id) administered cells and the iv injected cells were only able to induce IFN-y production by T and NK cells.^14^ However, in this study, id injected moDCs were unable to migrate to peripheral lymph nodes invalidating the comparison.^14^ Despite the different tissue localization of iNKT cell (subsets), other reports demonstrate activation of iNKT cells irrespective of their route of administration.^15-19^ These variable results with iNKT cell agonists and a lack of knowledge in the nanoparticle co-delivery setting prompted us to directly compare the effects of administration route on T and B cell responses against the co-delivered antigen. Even though iNKT cell frequencies are generally lower in humans, we trust that the similarity of iNKT cell tissue distribution between human and mice would substantiate the biologic relevance of our results.^20^

In this study, we used PLGA nanoparticles as a delivery system for antigen (Ovalbumin) and iNKT cell agonist (IMM60- threitolceramide 6). Our findings demonstrate that 1) Intravenous injection is the preferred route of administration for the iNKT cell-activating nanovaccines, and both intranodal or subcutaneously administered nanovaccines can hardly reach iNKT cell-rich lymphoid tissues to activate them. 2) More robust Type-I associated T and B cell responses can be measured after intravenous administration, 3) intravenous administration of PLGA nanovaccines is safe and do not cause any toxicity up to 50 mg/kg dose, 4) intravenously applied iNKT cell-activating nanovaccines can synergize with immune checkpoint modulation, PD-1 blockade and 4-1BB stimulation, in controlling tumor growth.

## Materials and methods

### Reagents and antibodies

PLGA (Resomer RG 502 H, lactide/glycolide molar ratio 48:52 to 52:48) was purchased from Boehringer Ingelheim. Solvents for PLGA preparation (dichloromethane) were obtained from Merck. CryoSure-DMSO from WAK-Chemie. Polyvinyl alcohol (PVA), isopropyl alcohol (IPA, ≥ 99.7%), water for HPLC (H_2_O), acetonitrile for HPLC (ACN, ≥ 99.9%), methanol for HPLC (MeOH, ≥ 99.9%) and anhydrous chloroform (CHCl_3_, ≥99%) were obtained from Sigma-Aldrich. Endotoxin-free ovalbumin (OVA) from Hyglos. OVA (257–264) SIINFEKL and HPV16 E7(49–57) were obtained from Anaspec. IMM-60 was kindly gifted by Ian Walters at IOX Therapeutics. Vivotag-S 750 fluorescent tag was purchased from Perkin Elmer and RPMI 1640 medium from Life Technologies Inc. CD3 (145-2C11) was obtained from BD, CD45.1 (A20), CD8α (53–6.7), XCR-1 (ZET), NK1.1 (PK136), CD11 c (N418), CD11b (M1/70), CD40 (3/23), I-A/I-E (M5/114.15.2), CD69 (H1.2F3), CD194-CCR4 (2G12), PD-1 (29 F.1A12) and CD90.1-Thy1.1 (OX-7), CD107a (1D4B), KLRG1 (2F1/KLRG1) antibodies were obtained from BioLegend. eBioscience™ Fixable Viability Dye eFluor™ 780 was purchased from Thermo Fisher. H2-Kb/SIINFEKL and CD1d- α-GalCer dextramers were purchased from Immudex. Celltrace CFSE, Celltrace- violet and Celltrace red were obtained from Invitrogen. For in vivo treatment, anti-PD-1 (RMP1-14), anti-PD-L1 (10 F.9G2), and anti-4-1BB (3H3) was obtained from BioXcell. HPV-16 E7 peptides RAHYNIVTFCCKCDS (LP) and RAHYNIVTF (SP) were obtained from Genscript.

### Nanoparticle synthesis

PLGA nanoparticles (NP) encapsulating only ovalbumin, NP (OVA); only IMM-60, NP (IMM60); a combination of both, NP(OVA+IMM60) or NP(HPV+IMM60) were prepared using a w/o/w emulsion and solvent evaporation–extraction method as described previously (10). Briefly, 100 mg of PLGA in 3 mL of dichloromethane containing 4 mg OVA or 2 mg HPV peptides and 80 µg IMM60 dissolved in DMSO was added to 25 mL of aqueous phase containing 2% polyvinyl alcohol and emulsified for 90 seconds using a digital probe sonicator (Branson Ultrasonics, Danbury, CT). The organic phase was evaporated overnight at 4°C and nanoparticles were collected by centrifugation at 14 000 rpm for 20 minutes, washed six times with ultrapure water and lyophilized.

In order to track nanoparticles (NPs) in vivo, the ovalbumin was labeled using a fluorescent dye with near-infrared emission (VivoTag S750, PerkinElmer) according to the manufacturer’s instructions. Briefly, 600 µL of 5 mg/mL of ovalbumin solution was diluted in 2400 µL of 50 mM carbonate/bicarbonate buffer. 100 µL of 1 mg/mL NHS-vivo750 dye solution (in DMSO) was mixed with ovalbumin solution and the mixture was incubated for 2 hours in dark under rotation. The unreacted dye was removed via several washing steps using 3 kDa spin filters (Millipore, Merck) and vivo750-labeled ovalbumin (OVA-vivo750) was lyophilized. Nanoparticles (NP) encapsulating labeled ovalbumin, NP (OVA-vivo750); and IMM60, NP (OVA-vivo750+ IMM60) were prepared using the same protocol as described above.

### Nanoparticle characterization

The size and polydispersity index of the nanoparticles was analyzed by dynamic light scattering using a Nanotrac Flex (Microtrac). The ovalbumin content of the NPs was determined using a Coomassie Plus Protein Assay Reagent (Pierce) according to the manufacturer’s protocol. The HPV peptide content was determined using a high-performance liquid chromatography (HPLC) system (Shimadzu, Kyoto, Japan) equipped with a Merck Chromolith RP-18e (100 mm × 4.6 mm) with a constant flow rate of 1 ml/min and detection at a wavelength of 220 nm. HPV peptide was extracted from nanoparticles by dissolving 1 mg nanoparticles in 100 μl DMSO followed by centrifugation at 14.000rpm for 10 minutes. 20 µL supernatant or standard was assayed with HPLC. The amount of HPV peptide was calculated by interpolation into the standard curves. IMM60 content of the NPs was determined by a Corona Veo Charged Aerosol Detector (CAD) coupled to an UltiMate 3000 high-performance liquid chromatography system (Thermo Fischer Scientific). The NPs were dissolved in CHCl_3_ for complete dissolution of the components following by the evaporation of the solvent and the end product was dissolved in IPA: H_2_O mixture (90:10) and analyzed by CAD on an XSelect CSH C_18_ 2.5 μm 3.0 × 150 mm XP column (Waters), eluents H_2_O-ACN-MeOH with ACN-MeOH gradient 0–100 vol. %, flow rate = 1.0 mL · min^−1^. The quantity of IMM60 was calculated by interpolation of the standard calibration curves of IMM60 performed in the same way as for the NPs. Nanoparticle characteristics are demonstrated in Table 1.

**Table 1.** Characteristics and contents of PLGA nanoparticles used in the study

Name	Size (nm)	SD (nm)	Antigen (µg/mg NP)	IMM60 content (ng/mg NP)	NP Dose (mg)/mouse
NP (OVA-vivo750)	211	60	42	N.A.	1
NP (OVA-vivo750+ IMM60)	205	65	56	118 ± 3	0.74
NP (OVA)	236	83	35	N.A.	0.187–0.0187
NP (OVA+ IMM60)	232	66	39	162 ± 10	0.172–0.0172
NP (IMM60)	176	47	N.A.	1080 ± 38	0.2
NP(HPV-16 E7 SP+IMM60)	171	38	14,9	268	0.9

### Mice and tissue

Wild-type C57BL/6 J, OT-I (C57BL/6-Tg (TcraTcrb)1100Mjb/Crl) and OT-II C57BL/6-(Tg(TcraTcrb)425 Cbn/Crl), CD45.1 (B6.SJL-PtprcaPepcb/BoyCrl), B6 Albino (B6 N-Tyrc-Brd/BrdCrCrl) and BALB/cAnNCrl mice were obtained from Charles River, Germany. Mice were aged 8–12 weeks at the start of experiments. Mice were maintained under specific pathogen-free conditions at the Central Animal Laboratory (Nijmegen, the Netherlands). Drinking water and food were provided ad libitum. Spleens, lymph nodes, and liver tissue were isolated under sterile conditions and stored at 4°C in RPMI 1640 medium supplemented with 100 U/mL penicillin and 100 μg/mL streptomycin until processing for maximally 2 hours. Spleens and lymph nodes were meshed through a 100 µm cell strainer by using a syringe plunger. The cell suspension was spun at 400xg for 5 minutes and resuspended in 3 mL of 1 x ammonium chloride solution for the lysis of erythrocytes. After 5 minutes of incubation at room temperature, cells were washed with 10 mL of PBS. Cells were counted by a hemacytometer. Liver tissue is processed as described in (26).

### Injections

For intravenous injections (iv), mice were warmed either in a heating chamber or under a heating lamp. All the nanoparticles, free antigens or transferred cells were delivered in 200 µL PBS solution through a lateral tail vein by a 1 mL syringe with a 29 G needle.

For subcutaneous injections (sc), under isofluorane anesthesia, nanoparticles or free antigen were delivered in 50 µL PBS solution on the right lateral tail base by a BD Micro-fine + insulin syringe 0.5 mL 30 G. The injection site was briefly massaged to facilitate draining.

For intranodal injections (inod), under isofluorane anesthesia, nanoparticles or free antigen were delivered in 10 µL PBS solution in the right inguinal (subiliac) lymph node by a BD Micro-fine + insulin syringe 0,5 mL 30 G as described in (27). Briefly, a 1–2 cm skin incision was performed, the lymph node was exposed with surgical tweezers followed by injection. Skin wound was closed with either suturing, clips or tissue adhesive (3 M Vetbond).

### In vivo imaging

B6 Albino (B6 N-Tyrc-Brd/BrdCrCrl) or BALB/c (BALB/cAnNCrl) mice were injected with either NP(OVA-vivotag750) or NP (OVA-vivotag750 + IMM60) containing 42 µg OVA-vivotag750 or 42 µg OVA-vivotag750 in solution via iv, sc or inod routes. Mice were shaved and imaged 0.5, 3, 24, 48, 72 and 96 hours after injections using an IVIS Lumina II (Perkin Elmer) system. Mice were euthanized and organs were dissected and imaged separately at different time points between 3 and 96 hours. The following imaging settings were used; Exposure time: 3 sec, Binning: Medium, F/Stop:2, Fluorescent Excitation filter:745 nm, Fluorescent Emission filter: 810–885 nm. A fluorescent background acquisition was performed for each time point. Living Image software (Caliper Lifesciences) was used for data analysis. Background values were subtracted from measurement values. Same sized ROI’s were applied on liver, bladder and injection sites for full-body image analysis, also same sized ROI’s were applied on the isolated liver, spleen, both kidneys, lungs, brain, and heart. Total flux (photon/second) per each ROI was calculated. B6 albino and BALB/c mice demonstrated very similar distribution profiles, therefore, data obtained from both strains were combined.

### In vivo antigen persistence

14, 10, 5 or 1 day prior to T cell transfer, naive C57BL/6 J mice were injected with NP(OVA+IMM60) containing 6 µg OVA and 30 ng IMM60 via different routes. On day 0, CD8 T cells were isolated from OT-I and CD4 T cells were isolated from OT-II mice spleen and lymph node tissues by using mouse CD8a+ T Cell or CD4 + T Cell Isolation Kits (Miltenyi Biotec). T cells were stained with 10 µM Celltrace dye according to the product manual and 1.5 × 10^6^ T cells were transferred to host C57BL/6 J mice. 16 h later, host mice were administered with 230 µg S1P1 inhibitor SEW2871 intraperitoneally. 48 hours after T cell transfer, host mice were euthanized, spleen and draining LNs tissues were processed and cells were analyzed with a flow cytometer (FACSVerse, BD). Mean proliferation was calculated using the formula log2(Celltrace MFI of T cells from control mice/Celltrace MFI of T cells from vaccinated mice).

### In vivo endogenous T cell generation

Naive C57BL/6 J mice were injected with NP(OVA+IMM60) containing 6 µg OVA and 30 ng IMM60 via different routes. In checkpoint blockade experiments, 200ug anti-PD-1 or anti-PD-L1 antibodies were delivered intraperitoneally on the day of vaccination and repeated every 3 days until the end of the experiment. For cytotoxicity experiments, some mice were transferred with CD45.1 naïve mouse splenocytes as target cells on day 6. 50% of the target cells were pulsed with SIINFEKL peptide and stained with Celltrace violet, while the other 50% were pulsed with HPV peptide and stained with Cell trace Far-red. Mice were euthanized on day 7 and 14 spleen and lymph nodes were isolated, cell suspensions were stained with CD3, CD8, and Kb SIINFEKL dextramers to evaluate the percentage of OVA-specific CTLs; with CD45.1 to evaluate target cell-specific cytotoxicity; with CD3, NK1.1, CD1d-aGalCer dextramer, and KLRG1 to evaluate NK and iNKT cell numbers and activation. For ELISPOT assays, splenocytes were used from mice that did not receive target cells.

### Ex-vivo cell culture

C57BL/6 J mice were injected with NP(IMM60) containing 216ng IMM60 via different routes. 3 hours later mice were euthanized and spleen and lymph nodes were processed. 1 × 10^6^ and 5 × 10^5^ total cells were immediately cultured in full RPMI1640 medium (supplemented with 10% FBS, 100 U/mL penicillin, 100 μg/mL streptomycin, 2 mM ultraglutamine and 50 µM 2-Mercaptoethanol) in 96 well plates. 24 hours later supernatants were collected for cytokine analysis by ELISA. Alternatively, varying numbers of total splenocytes, liver or lymph node cells were cultured in full RPMI1640 medium with or without 1 µg/mL IMM60. 24 hours later supernatants were collected for cytokine analysis by ELISA.

### ELISA/ELISPOT

Blood was collected via retro-orbital puncture during terminal anesthesia after indicated periods of time for each assay. Mouse anti-ova IgM, IgG1, IgG2 c, and IgE antibody assay (Chondrex) kits were used to determine serum antibody levels separately according to product protocols. All serum samples were diluted 1/100 for IgM, 1/1000 for IgG1, 1/250 for IgG2 c and 1/20 for IgE. IFN gamma, IL-4 or IL-17 Mouse Uncoated ELISA Kits (Invitrogen) were used to determine levels of each cytokine in serum or culture media according to product protocols. Serum samples were diluted 1/8 for IFN-γ ELISA. The culture medium was diluted 1/10 for IFN-γ, 1/2 for IL-4 and 1/5 for IL-17a. For ELISPOT, 2 × 10^5^ splenocytes were placed in a PVDF 96 well plate previously coated with anti–IFN-y antibodies according to the datasheet of the manufacturer (MABTECH, Mouse IFN-γ ELISpot BASIC (ALP)). Cells were stimulated with SIINFEKL peptide or left untreated. Average results of triplicate wells for each mouse splenocytes were reported.

### In vivo DC maturation and T cell activation

Naïve C57BL/6 mice were transferred with Celltrace-far red-stained 1.5 × 106 OT-I CD8 + T cells. Some mice did not receive any T cells as an internal control. One day later, some mice were injected with NP(OVA+IMM60) containing 6 µg OVA and 30ng IMM60, some mice with NP(OVA) containing 6 µg OVA via different routes. Some mice were left non-injected as a control. 20 hours later all mice were euthanized, liver, spleen and lymph nodes were isolated and processed. Only mechanical disruption, centrifugation, and erythrocyte lysis were performed during tissue processing. Isolated cells were stained with a viability marker and either a DC cocktail (CD11b, CD11 c, XCR1, MHC II, CD40), T cell cocktail (CD8, CD69, CCR4, PD-1, CD107a) and NK/B cell cocktail (NK1.1, CD19, CD69, CD3) at 4°C. Fresh samples were used in all analyses and samples were kept on ice during data acquisition. Data acquisition was performed on FACSVerse and FACSLyric flowcytometers. Data analysis was performed with Flowjo software. FCS files of all experiments and workspace files used for gating are available upon request.

### Tumor models

B16.ova (clone MO5) melanoma cell line was cultured in full RPMI1640 medium (supplemented with 10% FBS, 100 U/mL penicillin, 100 μg/mL streptomycin, 2 mM ultraglutamine, 1 mg/mL G418. 2,5x105 cells were reconstituted in 1/2 Matrigel/PBS and inoculated subcutaneously on the right flanks of naïve C57BL/6 mice. 9 or 10 days later mice were allocated in different treatment groups evenly so that each group has similar numbers of mice with similarly sized tumors (mean tumor size of 40–60 mm^3^). On the same day, vaccinations were performed with NP(OVA+IMM60) containing 6 µg OVA and 30 ng IMM60 or NP(OVA+IMM60) containing 0.6 µg OVA and 3 ng IMM60 via different routes. Depending on the assay, some mice were intraperitoneally injected with 200 µg anti-PD-1 (RMP1-14) 4 hours before the first vaccination and repeated every 3 days for 2 weeks. Tumor sizes were measured every other day with a caliper. Width x length x depth x 0.4 was used to calculate tumor volumes. Mice were euthanized immediately when tumor sizes exceeded 1500mm^3^, ulcerations were formed on tumors or the health of the mice was deteriorated. Blood was collected 1 day after nanoparticle vaccinations for IFN-y analysis and before euthanasia during terminal anesthesia for anti-ova antibody analysis.

TC-1 mouse lung adenocarcinoma cell line was transformed with E6 and E7 oncogenes of HPV-16 and the Ras oncogene. Cells were cultured in full RPMI1640 medium (supplemented with 10% FBS, 100 U/mL penicillin, 100 μg/mL streptomycin, 2 mM ultraglutamine, 0.4 mg/mL G418. 1 × 10^5^. Cells were reconstituted in 1/2 Matrigel/PBS and inoculated subcutaneously on the right flanks of naïve C57BL/6 mice. 10 days later mice were randomly allocated in different groups which have similar numbers of mice with similarly sized tumors (mean tumor size of groups 83mm^3^, SD: 9.8). Depending on the groups, mice were intraperitoneally injected with 200 µg anti-PD-1 (RMP1-14), 200 µg anti-PD-L1 (10 F.9G2), or 300 µg anti-4-1BB (3H3). 4 hours later, vaccinations were performed with NP(HPV+IMM60) nanoparticles. Vaccinations were repeated 7 days later once and antibody treatments were repeated every 3 days 4 times. containing 12 nmol (13,44 µg) HPV SP peptide and 240 ng IMM60 or 1.4 nmol HPV LP peptide and 28ng IMM60. Tumor sizes were measured every other day with a caliper. Mice were euthanized immediately when tumor sizes exceeded 2000mm^3^, ulcerations were formed on tumors or the health of the mice was deteriorated. Blood was collected 1 day after nanoparticle vaccinations for IFN-y analysis.

### Statistical analysis

One-way or Two-way ANOVA followed by Uncorrected Fisher’s LSD was used for Gaussian distributed data, Kruskal-Wallis followed by uncorrected Dunn’s test was performed if normality tests fail. Survival analyses were performed by Log Rank (Mantle Cox) test. GraphPad Prism version 8.1.0 for Windows was used for all statistical analysis and figures. *, *P* < .05; **, *P* < .01; ***, *P* < .001; and ****, *P* < .0001. ns, not significant. Mice were randomly assigned to all experimental groups (except B16.ova tumor experiments) based on online randomization software. Blinding was not possible during injections due to different routes applied. Tumor size measurements, antibody injections, and blood collections were performed in a blinded manner by bio technicians.

## Results

### Biodistribution of intravenous, subcutaneous and intranodal administered nanovaccine

**Figure 1.**
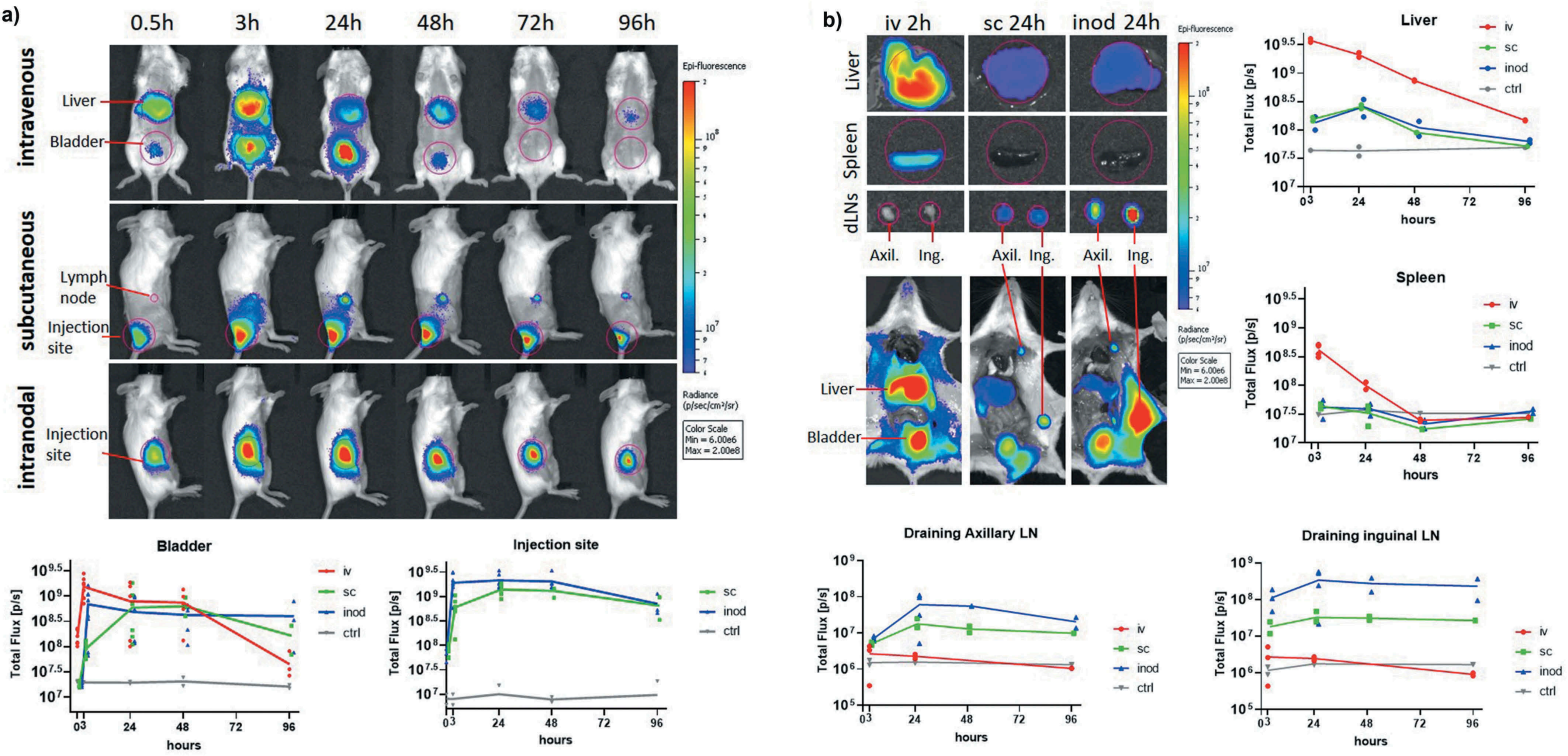
Kinetics of antigen distribution delivered by nanoparticles. Kinetic analysis of Ovalbumin-vivotag750 distribution was performed to compare main antigen drainage sites and timings via different injection routes. Wild-type BALB/c mice were injected with 1 mg of PLGA nanoparticles containing 41.7ug Ovalbumin-vivotag750 via intravenous (iv), subcutaneous (sc) or intranodal (inod) routes. (a) Full-body images were acquired along with one uninjected control mouse. The pseudo-color image of the fluorescence was overlaid on the photograph. Same sized regions of interest (ROI) were selected (red circles) and fluorescence was quantified in each ROI. Ventral images were used to quantify liver and bladder fluorescence. (b) Mice were euthanized at different time points, images of excised organs were acquired separately and fluorescence was quantified in each ROI (same ROI size was applied for Liver and spleen). Each mouse is depicted as a dot with mean values of groups, N = 2–8 per time point per route. (iv) red, (sc) green, (inod) blue

The biodistribution of PLGA-based nanovaccines has not been extensively explored, let alone direct comparison of different routes of administration. Here, we evaluated antigen drainage at various time intervals (0.5–96 hours) by using fluorescently labeled antigen (ovalbumin-Vivo750) encapsulated in PLGA nanoparticles. Already 30 minutes after intravenous injection, OVA-Vivo750 could be detected in the liver and in the bladder via whole-body imaging (Figure 1a). By contrast, subcutaneous and intranodal administration led to substantially lower drainage to the liver, and most of the antigen remained at the injection site (Figure 1a). In addition, visceral organs were excised and imaged individually at different time points. After intravenous injection, OVA-Vivo750 was highest in the liver, followed by spleen where fluorescence dropped to background levels within 48 hours (Figure 1b). In contrast, subcutaneous and intranodal administration delivered less antigen to the liver but more to the lymph nodes (LNs) where antigen was stably retained for at least four days (Figure 1b). As expected, intranodal administration was more efficient in antigen delivery to LN than subcutaneous administration (Figure 1b). Hardly any antigen could be detected in the spleen after subcutaneous and intranodal administrations, and likewise, no significant drainage to the lymph nodes was detected after intravenous administration.

In order to evaluate how encapsulation of antigen within nanoparticle formulations affected its biodistribution after intravenous administration, the free antigen was compared with PLGA nanoparticles encapsulating the antigen. Free antigen displayed a more ubiquitous distribution over all tissues whereas nanoparticles favored accumulating in the liver and spleen (Sup.fig1-A-B). As a result, nanoparticles were subjected to slower systemic clearance as demonstrated by lower kidney fluorescence (Sup.fig1B). Nanoparticles followed a similar distribution pattern irrespective of their IMM60 content. Of note, we did not observe any mortality or side effects in any of the 18 intravenous injected mice that received the highest dose of nanoparticles in this study (1 mg/mouse). Moreover, overall serum alanine transaminase activity was low (200-300UI) compared to toxic levels observed in aged mice (>3000UI)^21^ (Sup.fig1 C).

**Figure 2.**
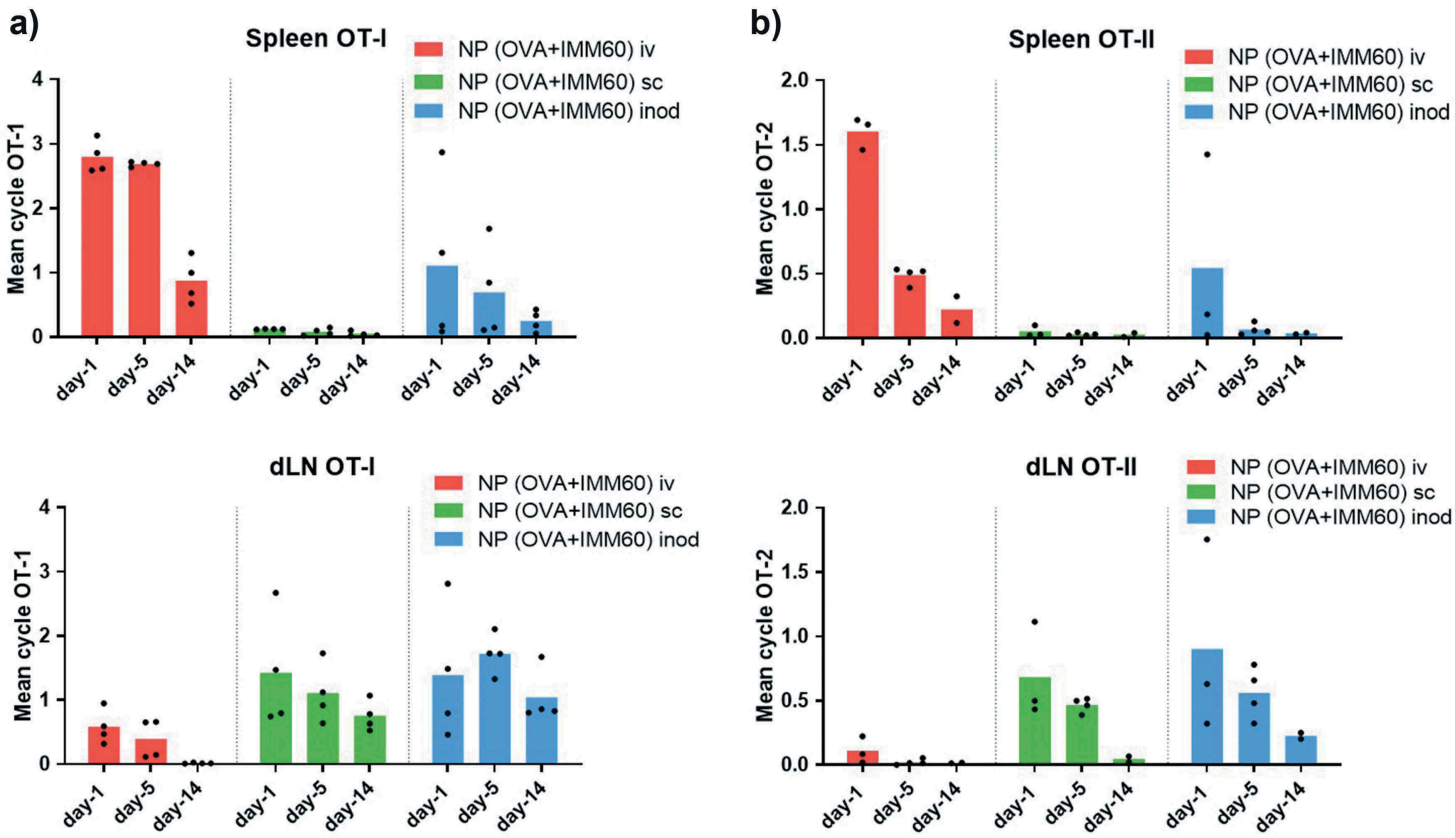
Retention of antigen presentation. Kinetics of antigen presentation was compared for a two week period after different routes of injection. Wild-type C57Bl/6 mice were injected with 0,172 mg of PLGA nanoparticles (containing 6ug Ovalbumin and 30ng IMM60) 14, 10, 5 or 1 day before ovalbumin-specific CD8+ (OT-I) and CD4+ (OT-II) T cell transfer. Injections were performed via intravenous (iv) red, subcutaneous (sc) green or intranodal (inod) blue routes, 48 hours later, the proliferation of (a) OT-I T cells, and (b) OT-II T cells were assessed on the spleen and draining lymph nodes. N = 4 mice per each time point and route. The mean cycle of proliferation was calculated as explained in methods. Each mouse is depicted as a dot with mean values of groups

Although fluorescent imaging is valuable to follow particles at early time points, it is harder to assess the fate of antigen over prolonged periods of time. In particular, when intracellularly located ovalbumin gets processed entirely into peptides and the fluorescent label is released. To gather information on the long term biodistribution, we carried out *in vivo* T-cell proliferation assays following different injection routes of the nanovaccine. To this end, we administered the nanoparticles 1, 5, or 14 days before the transfer of OT-I and OT-II T cells and measured their proliferation two days later. The data confirmed the main antigen drainage sites: spleen after intravenous injection and inguinal lymph nodes after intranodal and subcutaneous injections (Figure 2a). Given the high sensitivity of this technique, we were able to detect small quantities of antigen in the spleen after some of the intranodal injections, and in the LNs of intravenously injected mice at early time points. In contrast, no antigen presentation occurred in the spleen after subcutaneous injections. (Figure 2a). OT-II CD4^+^ T cell proliferation exhibited a similar antigen drainage profile. However, antigen presentation via MHC class II demonstrated a more rapid decline for all injection routes (Figure 2b). In conclusion, antigen mainly accumulated in liver and spleen after intravenous injection, and in draining lymph nodes after intranodal and subcutaneous administration.

### Intravenous administration results in more consistent cytotoxic T cell and B cell-mediated immunity

**Figure 3.**
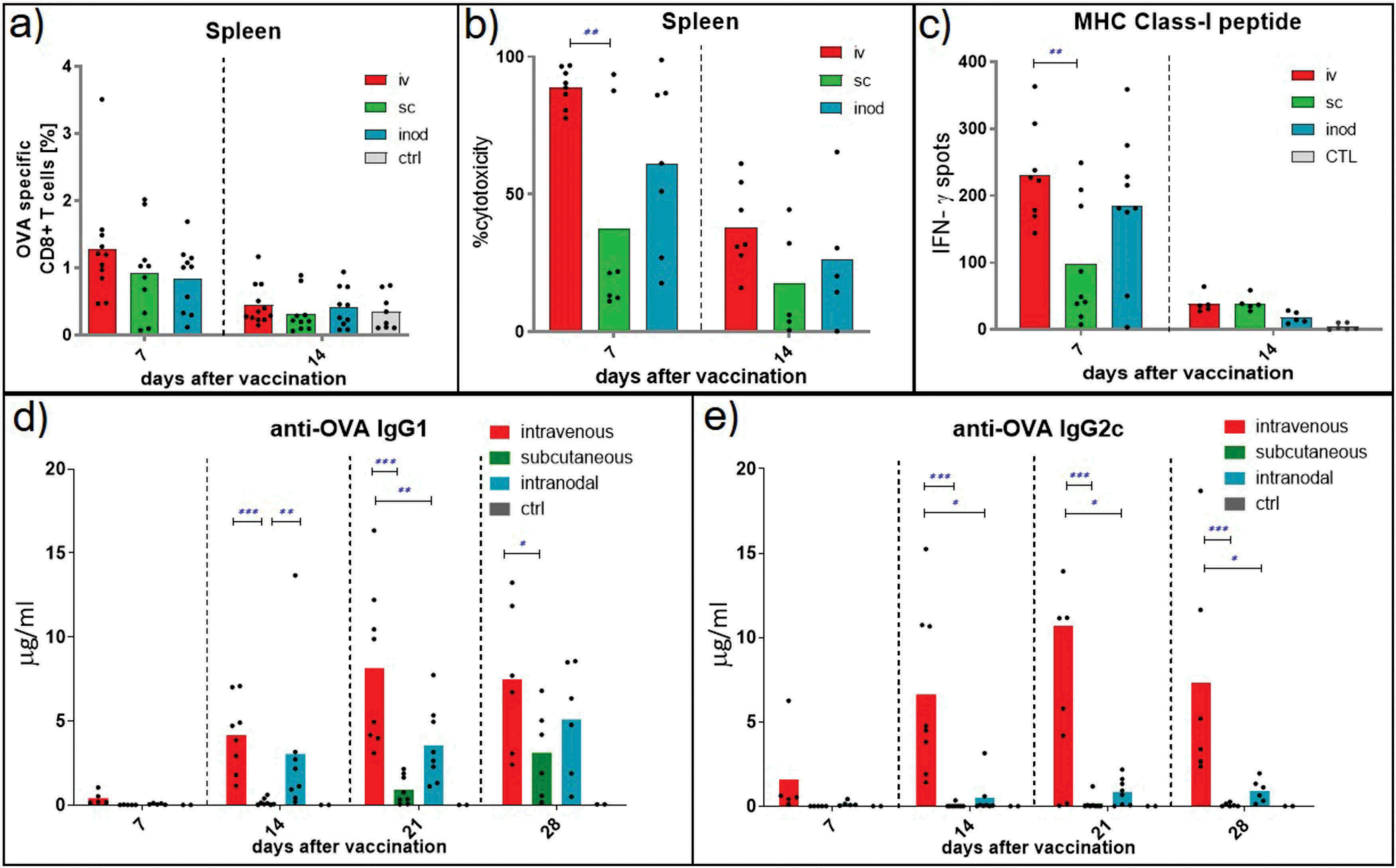
Endogenous T and B cell responses. Kinetics of endogenous T and B cell responses against ovalbumin was compared after different routes of injection. Wild-type C57BL/6 mice were injected with 0,172 mg of PLGA nanoparticles (containing 6ug Ovalbumin and 30ng IMM60) via different routes. 7, 14, 21 and 28 days later, mice were euthanized, serum and spleens were isolated. (a) Ovalbumin-specific CD8 + T cells were analyzed by fluorescent H-2K^b^-SIINFEKL Dextramer binding. % of specific cells to all CD8 T cells were demonstrated. N = 10–12 mice per group. (b) Some of the mice were transferred with ovalbumin peptide-loaded target cells on days 6 and 13 after vaccinations. Antigen-specific toxicity is shown as % of target cells killed in host 1 day after transfer. N = 5–8 mice per group. (c) Splenocytes were isolated from the mice that did not receive target cells. The same number of cells were incubated with ovalbumin derived peptides [MHC class I binding *SIINFEKL]* (c) on 96-well IFN-γ ELISPOT plates for 24 hours. The mean value of three wells per mouse is demonstrated. N = 5–9 mice per group. (d, e) Serum samples isolated at indicated time points were evaluated for IgG1 (d) and IgG2 c (e) anti-ovalbumin antibodies by ELISA. N = 5–8 mice per group. Each mouse is depicted as a dot with mean values of groups.(a,b,c) Unpaired t-test with Welch’s correction; (d,e) Two-way ANOVA was used for statistical analysis

Next, we determined whether the generation of an endogenous ovalbumin-specific T cell response was affected by the route of nanovaccine administration. We observed no significant difference in the number of antigen-specific CD8^+^ T cells in the spleens between groups of mice vaccinated via different routes. On day 14, when the immune response was declining, the number of antigen-specific CD8^+^ T cells also contracted similarly in all groups (Figure 3a). By contrast, we observed a significantly higher in vivo, antigen-specific, cytotoxicity by intravenous vaccinations on day 7 (Figure 3b). A similar observation was made when we re-stimulated splenocytes with SIINFEKL peptides in an Elispot assay. The number of IFN-y producing CD8^+^ T cells was significantly higher in mice that were injected intravenously than the mice injected subcutaneously on day 7 (Figure 3c).

B cell responses also require T cell help, which plays a critical role in antibody isotype switching. It has been reported that iNKT cells can also directly or indirectly provide help to the B cell responses.^22^ Measuring the serum levels of ovalbumin-specific antibodies revealed a faster emergence of the Th2 linked anti-ovalbumin IgG1 antibodies after intravenous or intranodal administration when compared to the subcutaneous route (Figure 3d). The most striking difference was observed in Th1 associated IgG2 c switching, which appeared to be mostly restricted to the intravenous route of administration (Figure 3e). In summary, nanovaccine administration via intravenous route resulted in higher cytotoxicity and Th1 type help than the subcutaneous route, while in particular intravenous injection supported B cell responses to a greater extent.

### Route of administration affects cytokine profiles, depending on iNKT cell subsets at the target organ

**Figure 4.**
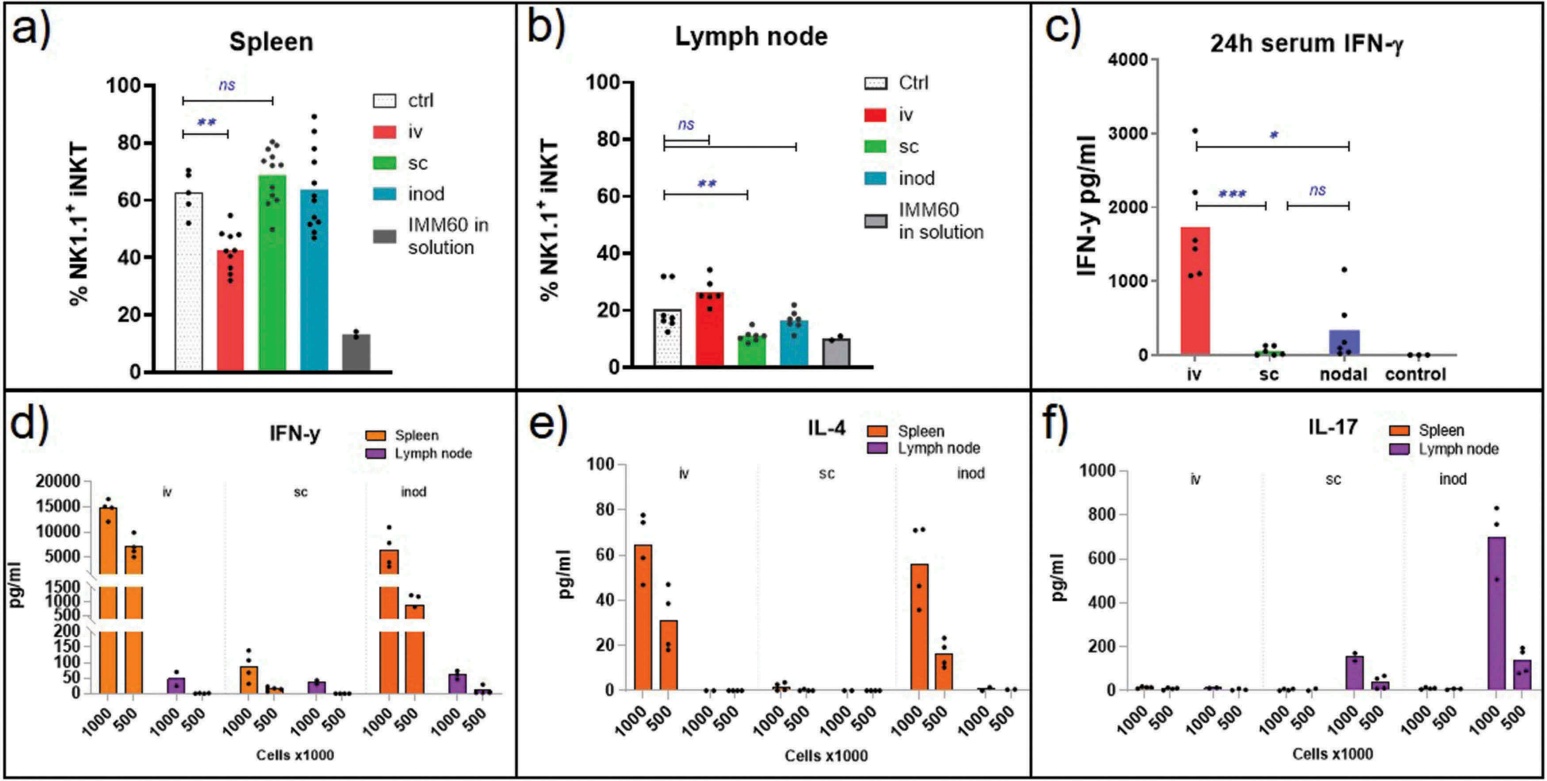
Evaluation of prevalent iNKT cell subsets in spleen and lymph nodes. (a, b, c) Wild-type C57BL/6 mice were injected with 0,172 mg of PLGA nanoparticles (containing 6ug Ovalbumin and 30ng IMM60) via different routes or 1ug IMM60 in solution via the intravenous route. (a, b) NK1.1 positive percentage of the spleen and lymph node iNKT cells were analyzed 7 days after injection of OVA+IMM60 nanoparticles. N = 10–12 mice per group (a), N = 6–7 mice per group (b). An unpaired t-test with Welch’s correction was used. (c) Serum IFN-γ levels were analyzed 24 hours after injections. N = 6 mice per group (d, e, f) Wild-type C57BL/6 mice were injected with 0,2 mg of PLGA nanoparticles (containing 216ng IMM60) via different routes. Spleen and lymph nodes were isolated 3 hours after injections and 10^6^ or 5 × 10^5^ total cells were incubated for 24 hours. Culture supernatants were analyzed for IFN-γ, IL-4 and IL17A levels by ELISA

In order to assess the level of iNKT cell activation via different vaccination routes, we first evaluated the down-modulation of NK1.1 expression on iNKT cells. Even though the NK.1.1^+^ fraction of iNKT cells varies in different organs, down-modulation of NK1.1 upon stimulation is a well-documented observation indicative of activation.^23,24^ As a positive control, we injected a high dose (1 µg) of free IMM60 intravenously. This reduced the NK1.1^+^ fraction of iNKT cells both in the spleen and lymph nodes within 7 days (Figure 4a). Intravenous injection of (OVA+IMM60) nanoparticles also resulted in significant activation of iNKT cells, visible in the spleen as a reduction of the NK1.1^+^ fraction. However, subcutaneous or intranodal injection of the same nanoparticles had no major impact on NK1.1^+^ fractions of iNKT cells in the spleen (Figure 4a).

In the lymph node, the NK1.1^+^ subset was less prevalent than in spleen (Figure 4b), and a significant reduction was only apparent after subcutaneous administration. NK1.1 positive iNKT cells are considered to be comprised of mainly iNKT1 cells that can secrete IFN-γ upon stimulation.^2^ Therefore, we measured the serum IFN-γ levels 24 hours after injection to determine the contribution of iNKT1 cells. IFN-γ levels were highest after intravenous injection (Figure 4c). However, subcutaneous injections, in spite of the evident NK1.1 downregulation in the lymph nodes, resulted only in minimal IFN-γ levels in the serum (Figure 4b,c). To investigate the origin of IFN-γ in more detail, spleens and lymph nodes were isolated 3 hours after NP(IMM60) administration. Subsequently, spleen and lymph node-derived cells were cultured ex–vivo for 24 hours to detect cytokines associated with iNKT cell activation. As expected, intravenous injection induced the major IFN-γ production from spleen cells (Figurer 4d). However, intranodal injection also induced considerable IFN-γ production from iNKT cells residing in the spleen but not from those in the lymph node (Figure 4d). Interestingly, when checked for other iNKT cell-associated cytokines, spleen and the lymph node-derived cells demonstrated a completely different cytokine profile after IMM60 stimulation. While both IFN-γ and IL-4 was produced in spleen and liver, IL-17A was strongly induced in the lymph nodes (Figure 4d–f; Supp fig 2-A, B, C). These results demonstrate that peripheral lymph nodes do not contribute to systemic IFN-γ production and intranodal injection can only slightly contribute due to sporadic drainage to other organs. Results also confirm that subcutaneous and intranodal injections can activate lymph node-resident iNKT cells, and, IL-17A response is prominent in this compartment.

#### iNKT cell help after vaccination via different routes

**Figure 5.**
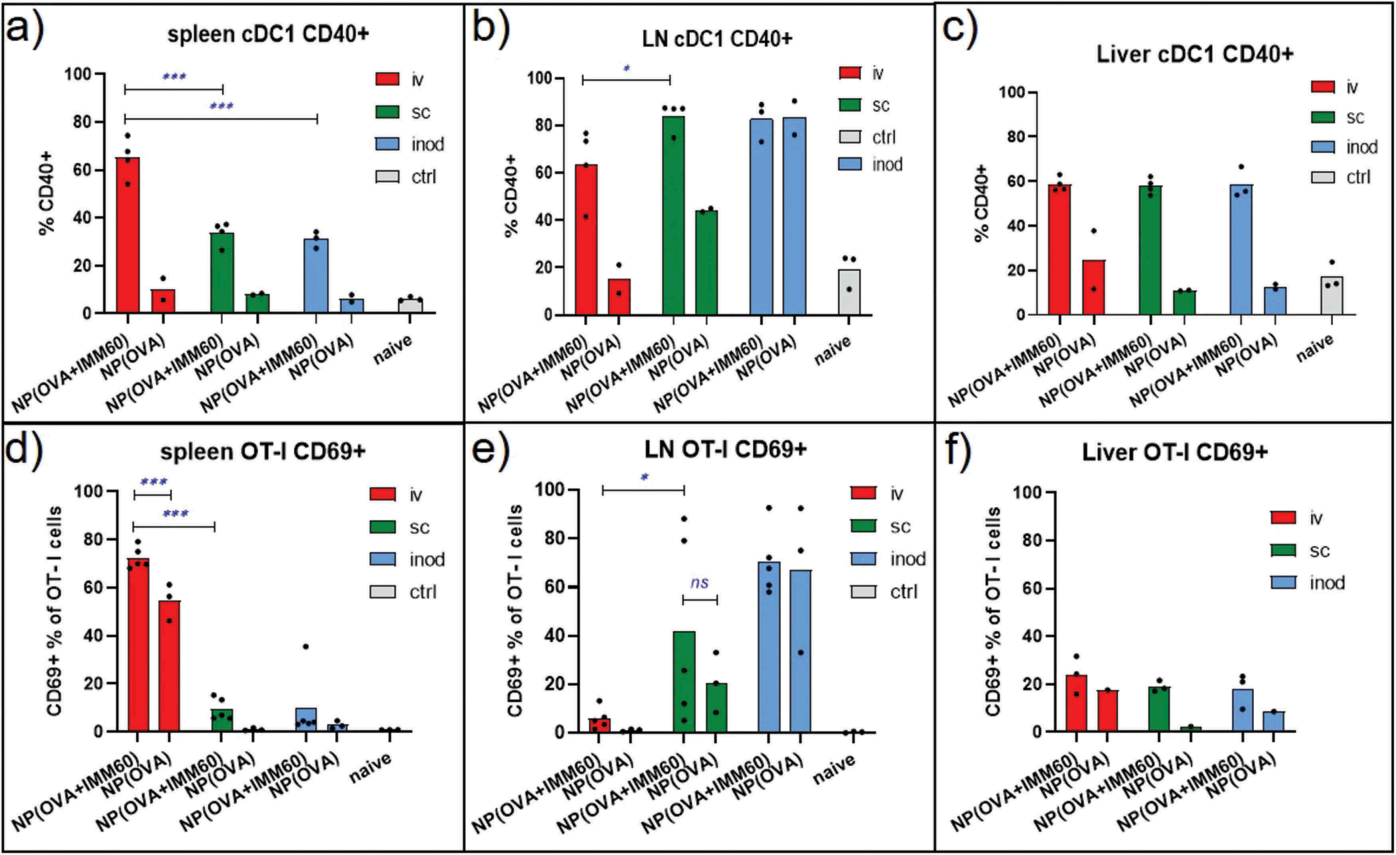
iNKT cell help in the lymph node. Wild-type C57BL/6 mice were transferred with OT-I T cells and one day later injected with 0,172 mg of PLGA nanoparticles (containing 6ug Ovalbumin and 30ng IMM60) via different routes. 24 hours later spleens and lymph nodes were isolated. (a–c) CD40+ percentages of XCR-1+ cDC1 s in (a) spleen, (b) lymph nodes and (c) livers. (d–f) OT-I T cells were analyzed for CD69 expression in (d) spleen, (e) lymph nodes, (f) livers. Each mouse is shown as a dot with mean values. Two-way ANOVA or Kruskal Wallis tests were used for statistical analysis

We assessed the CD40 expression on classical DC subsets as an indirect marker of iNKT cell activation and direct evidence for DC licensing. Despite the limited number of NKT1 cells in lymph nodes, substantial maturation of cDCs was observed in the lymph nodes 24 hours after subcutaneous injections as well as in the spleen after intravenous injections of IMM60 containing nanoparticles (Figure 5a, b, Supp.fig. 3A). Apart from these main drainage regions, an increase of CD40 positivity was also detectable within XCR-1+ cDC1 populations but not in cDC2 populations at non-draining areas (spleen for subcutaneous and lymph nodes for iv injections) (Figure 5a,b, Supp.fig. 3A). Of note, we observed a spontaneous maturation of DCs and activation of T cells in the lymph nodes after intranodal injections independent of nanovaccines possibly due to surgery-mediated local inflammation (Figure 5b,e; Supp.Fig.3B). Regarding the liver XCR-1+ cDC1 s, we observed similar iNKT cell-mediated maturation independent of the route of administration of the nanovaccine (Figure 5c) (Figure 5).

Next, we measured the CD69 upregulation on transferred OT-I T cells as evidence for iNKT cell help to T cell priming. We found that CD69 upregulation on splenic OT-I T cells is mostly restricted to the intravenous injection route (Figure 5d). PD-1 and CD107a upregulations on spleen OT-I T cells were also highly restricted to intravenous injections of IMM60 containing particles (Sup.Fig.3 C-D). CCR3 upregulation on splenic OT-I T cells was higher after iv injections than subcutaneous but some intranodal injected mice also demonstrated an increase (Sup.fig. 3E).In the lymph nodes, there was no consistent upregulation of CCR4, CD69 or PD-1 on OT-I T cells due to injection route or iMM60 content (Figure 5e, Supp.fig.3 C, E). Although, some mice demonstrated elevated levels of CD69 and PD-1 after subcutaneous and intranodal injections respectively and CD107a expression was restricted to intranodal injections in the lymph nodes (Sup.Fig-3D). Regarding the liver OT-I T cells, we could not observe a major increase of CD69 irrespective of the route of administration (Figure 5f).

Next, we checked the bystander effects of iNKT cell activation to other lymphocytes in different organs. We observed that IMM60 containing nanoparticles induce CD69 upregulation on host CD8^+^ T cells, B cells, NK cells and NKT cells in the spleen, lymph node and liver (Sup.Fig-4). Similar to the activation of DCs and OT-I T cells, the above-mentioned lymphocytes in the spleen were mostly activated by iv injection while intranodal injections mostly activate the ones in the injected lymph node. Lymphocytes isolated from the liver did not demonstrate a preferential route of administration (Sup.Fig-4A,B,C). Total NKT cells (defined as CD3+ and NK1.1+ cells) in spleen and liver also demonstrated similar upregulation of CD69 after different routes of injections (Sup.Fig-4D). The CD69 upregulation was previously reported on various bystander cells after iNKT cell agonist applications and shown to be dependent on high levels of cytokines such as IL-4 and IFN-γ.^25,26^ In summary, we observed that iNKT cell activation contributes to DC maturation and activation of co-delivered antigen-specific T cells or other bystander lymphocytes in the lymph node, spleen, and liver.

### Route of administration and tumor growth control

**Figure 6.**
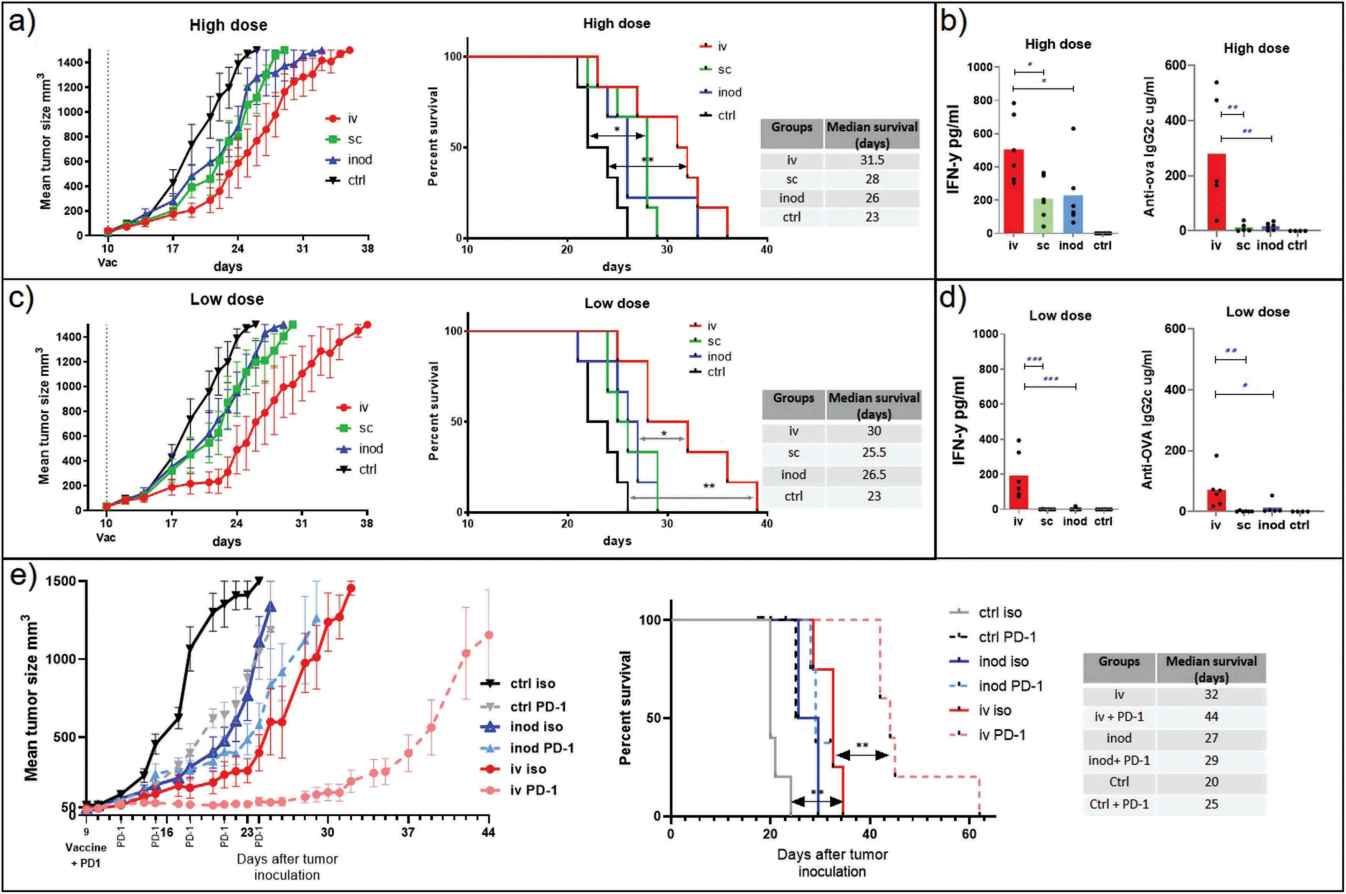
Effect of therapeutic vaccination route on tumor growth. We compared the efficacy of two doses of therapeutic vaccination on control of B16.ova melanoma growth. Mice were inoculated with tumor cells on the right flanks. 9–10 days later injected via different routes with high dose 0,172 mg of PLGA nanoparticles (containing 6ug Ovalbumin and 28ng IMM60) (a, b), or low dose 0.0172 mg PLGA nanoparticles (containing 0.6ug Ovalbumin and 2.8ng IMM60) (c, d, e). Subcutaneous and intranodal vaccinations were performed on the same side of the animal as the tumors inoculated. (a) Tumor growth and survival curves of high dose treatment are shown. Log-rank (Mantel-Cox) test was used to compare survival curves. (b) 24-hour serum IFN levels and endpoint serum anti-ovalbumin IgG2 c levels of high dose treatment in A were shown. One-way ANOVA was used for comparisons. N = 6 mice per group (c, d) As in A and B but a low dose of particles was used. N = 6 mice per group. (e) As in C, a low dose of particles was introduced via intravenous or intranodal routes on day 9 after tumor inoculation. Some mice received anti-PD-1 antibodies intraperitoneally starting on day 9 before particle injections and repeated every 3 days for 2 weeks. N = 5 mice per group. Log-rank (Mantel-Cox) test was used to compare survival curves

Intravenous administration of nanovaccines induced more potent cytotoxic T cells in naïve mice (Figure 3b). However, this may not hold true for tumor-bearing mice where T cells are primed by tumor-derived antigens in advance. Moreover, the outcome of subcutaneous and intranodal injections may be affected by the proximity of the tumor or by tumor-derived factors. Therefore, we tested the three routes of administration in tumor-bearing mice. Our results demonstrated that intravenous vaccination is most effective in delaying tumor growth and the induction of systemic IFN-γ levels (Figure 6a,b). Even though IFN- γ levels were also elevated in tumor-bearing mice injected subcutaneously, anti-ova IgG2 c production was only found after intravenous vaccination (Figure 6b). We have previously reported anti-tumor response with iv injections of 0.6 µg ovalbumin containing NP(OVA+aGalCer) particles.^8^ Therefore, we reduced the dose 10 times to reproduce these results with different administration routes. At a low dose, the survival benefit of the nanoparticle delivery by the intravenous route became even more prominent over intranodal and subcutaneous routes, in which the systemic IFN-γ has disappeared (Figure 6c, d) (Figure 6).

### iNKT cell-activating nanovaccines in combination with immune checkpoint antibody treatment

The tumor growth curves showed a steep increase around day 14 after vaccination, which overlapped with the period when the contraction of T cells occurred (Figure 3a–c). T cell contraction is known to be dependent on signaling by the immune checkpoint molecule PD-1 and we also observed a prominent PD-1 upregulation on T cells 24 hours after vaccination (Supp.Fig-3D). Therefore, we questioned whether the inhibition of tumor growth could be extended by blocking the PD-1 signaling. We compared the tumor growth in mice treated with anti-PD-1 antibody or isotype antibody and vaccinated either via intravenous or intranodal routes. The subcutaneous route was omitted based on previous poor performance in assays testing the induction of cytotoxic T cell responses, anti-ova antibody, and IFN-γ productions, antigen-specific T cell, and bystander lymphocyte activation levels. Indeed, we observed a clear increase in the delay of tumor growth when vaccination is combined with anti PD1 blocking antibodies. Surprisingly, of the groups treated with the anti-PD-1 antibody, the most striking increase in survival was observed within the intravenously vaccinated mice group (Figure 6e). Intranodal vaccinated or non-vaccinated mice did not significantly benefit from the anti PD1 antibody treatment. Together, these findings show that the intravenous route of administration is most effective in controlling tumor growth. Importantly we show that this route of administration acts synergistically with immune checkpoint blockade while other routes of administration do not.

### Potential mechanisms of vaccination and checkpoint blockade synergy

**Figure 7.**
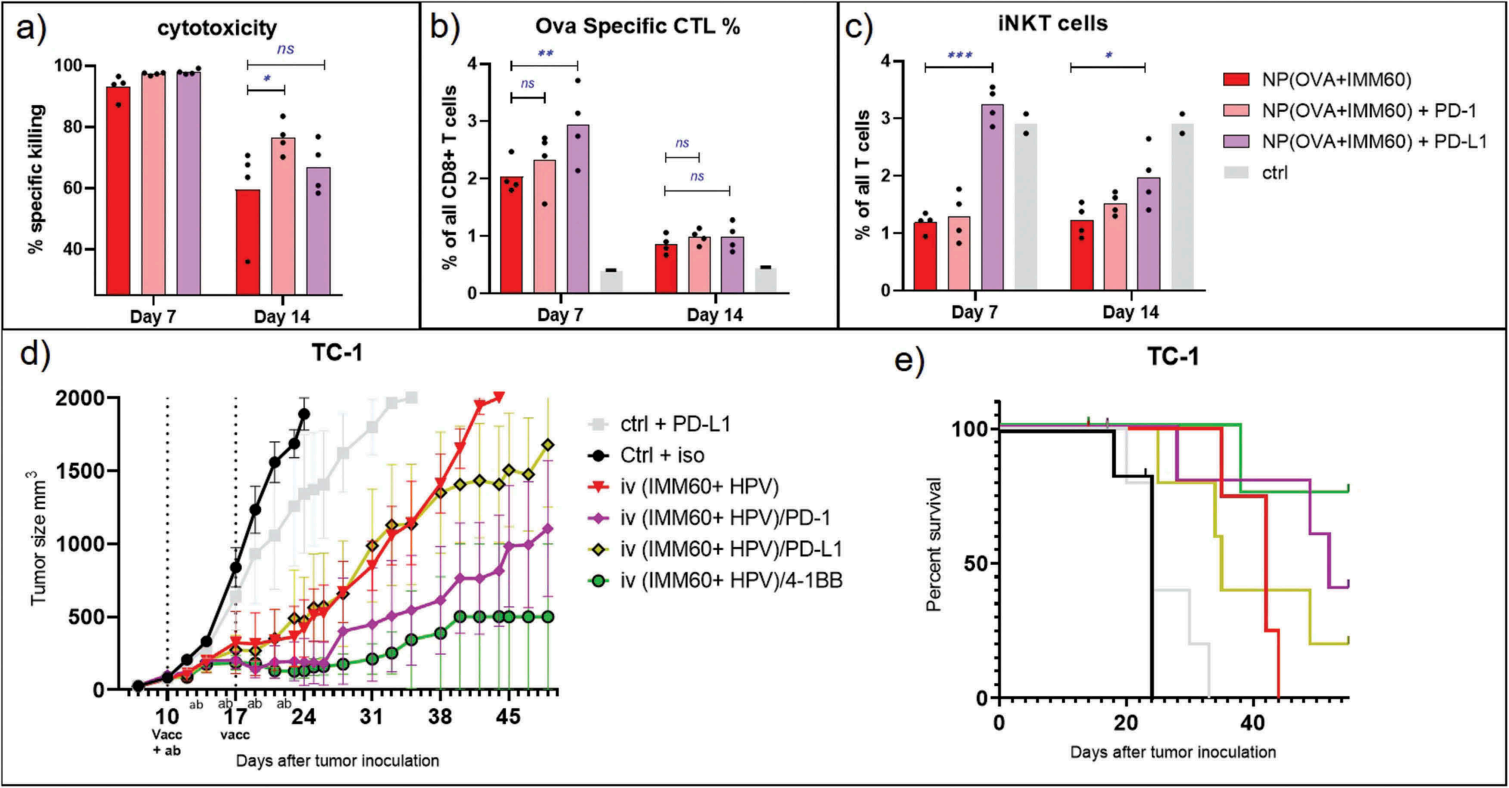
Combination therapy. Mechanism of checkpoint blockade therapy and application in another tumor model. Wild-type C57BL/6 mice were intravenously injected with 0,172 mg of PLGA nanoparticles (containing 6ug Ovalbumin and 30ng IMM60) with or without intraperitoneal PD-1 or PD-L1 injections repeated every 3 days. (a) Mice were transferred with ovalbumin peptide-loaded target cells on days 6 and 13 after vaccinations. Antigen-specific toxicity is shown as % of target cells killed in host 1 day after transfer. (b) Ovalbumin specific CD8 + T cells were analyzed by fluorescent H-2K^b^-SIINFEKL Dextramer binding. % of specific cells to all CD8 T cells were demonstrated. (c) iNKT cell percentages among all CD3 positive NK1.1 negative cells as determined by fluorescent CD1d-α-GalCer Dextramer binding. Mean values are shown with each dot representing the value of a mouse. (d–e) C57BL/6 mice were subcutaneously implanted with TC-1 tumor cells on day 0. 10 days later mice received antibodies intraperitoneally and vaccinated with nanoparticles containing 13 µg (12 nmol) HPV E7 peptide and 260ng IMM60. Antibody treatment was repeated four times every three days and vaccination was repeated one week later. (d)Mean tumor sizes of the treatment groups shown with standard errors. (e) Survival curves of the mice in D, censored points were plotted as dots. N = 6 mice per group

In order to test the hypothesis that checkpoint blockade combination might prevent T cell contraction as a potential mechanism for extended tumor control, we compared intravenous vaccination of naïve mice with the treatment of anti-PD-1 or anti-PD-L1 antibodies. 7 days after vaccinations, there was no distinctive cytotoxicity measurable because T cell responses were already saturated in mice not receiving any antibody treatment (Figure 7a). When checking the numbers of antigen-specific T cells, we observed that PD-L1 treatment resulted in higher numbers of ovalbumin specific T cells on day 7 (Figure 7b). 14 days after vaccination, reduced cytotoxicity could be observed in all experimental groups (Figure 7a). Comparing the different groups, we observed that PD-1 blockade induced a significantly higher cytotoxic potential than non-treated mice but PD-L1 blockade failed to reach the same levels at this time point (Figure 7a). Contraction of antigen-specific CD8^+^ T cells occurred similarly in all groups on day 14 invalidating the contraction theory (Figure 7b). Looking at the iNKT cells, anti-PD-L1 antibody treatment but not anti-PD-1 antibody treatment prevented their contraction which is otherwise present both on days 7 and 14 after vaccinations (Figure 7c). This difference with PD-L1 blockade was not due to lower activation of iNKT cells because these exhibited similar upregulation of KLRG1 when compared to other groups, and even further downmodulation of surface NK1.1 as an indication of iNKT cell activation (Sup.Fig-5A, B). On the other hand, the PD-L1 blockade resulted in a reduction of NK cell frequencies in the spleens of mice as observed both on 7 and 14 days after vaccinations but their activation levels were not affected (Sup.Fig-5C, D). In conclusion, we could demonstrate a prominent extension of cytotoxic functions of CTLs with PD-1 checkpoint blockade (Figure 7).

### Combination of nanoparticle vaccines and agonistic antibody treatment

Previously, Bartkowiak et.al. compared the synergism of HPV peptides and iNKT cell agonist α-GalCer vaccination with various checkpoint inhibitor or agonist antibodies and demonstrated an exceptional growth control of TC-1 tumors with anti-4-1BB antibody combination but not with anti-PD-1.^27^ In order to test if anti-4-1BB antibody treatment would also synergize with nanoparticle vaccines delivered via the iv route, we utilized the TC-1 mouse adenocarcinoma cell line which expresses the HPV E6 and E7 oncogenes as a relevant antigen for human cervical cancers. We produced PLGA nanoparticles containing the minimal class I MHC binding peptide (RAHYNIVTF). When we checked the 24 h systemic IFN-γ levels of nanoparticles containing HPV peptides and IMM60 NP(HPV+IMM60), we observed a significant increase with the anti-4-1BB combination but also a modest increase with anti-PD-1 combination (Sup.Fig-5E). Treating TC-1 tumors with NP(HPV+IMM60), we observed similar results as in B16.ova tumors (Figure 7d). In our experiments, both the combination of vaccination with anti-PD-1 or anti-4-1BB antibodies effectively delayed tumor growth (Figure 7e, Sup.Fig.-6B). By contrast, a combination of nanoparticle-mediated vaccination with PD-L1 blockade had only a limited effect on tumor growth compared to vaccination alone (Figure 7a, d). Much to our surprise, despite delaying the contraction of iNKT cells, PD-L1 treatment completely abrogated the IFN-γ production by iNKT cells in tumor-bearing mice after a second vaccination one week later (Sup.Fig.5E).

Taken together, we could demonstrate the synergy between intravenous nanoparticle vaccination and PD-1 blockade not only in the B16 ova model but also in the TC-1 tumor model. PD-L1 blockade appeared less effective and 4-1BB stimulation is more potent.

## Discussion

Given the fact that iNKT cells are mainly found in spleen and liver, it is logical to anticipate a higher impact upon intravenous (iv) administration as it was performed in multiple preclinical tumor models and early phase I clinical trials.^14,28-30^ Nevertheless, several groups also applied other administration routes and some claim to obtain better iNKT cell and NK cell activation than iv injections.^7,16-18,31-33^ Besides, the iNKT mediated help over antigen-specific T and B cell responses has not been compared in different immune compartments and administration routes. In this study, we aimed to provide a detailed insight into how iNKT cell agonists can be optimally delivered in nanoparticle formulations to get maximum iNKT cell contribution toward cancer immunotherapy.

We confirmed that nanoparticles were mainly delivered to the liver and spleen immediately after intravenous administration, while subcutaneous and intranodal injections drained mostly to local lymph nodes. We observed more potent Th1 type ovalbumin specific T cell and B cell responses after intravenous injections. This was predictable as a consistent and higher systemic IFN-γ was also visible after intravenous vaccinations. The reason for the higher IFN-γ production is both higher numbers of iNKT cells present in spleen and liver than peripheral lymph nodes and a higher percentage of iNKT1 subset (70–80%) among them.^34^ Moreover, it is known that NK cell transactivation by iNKT cells also contributes to IFN-γ levels and this effect is restricted to spleen and liver where NK cells are present but not in lymph nodes.^25,35^ The same is also relevant for the human body, where NK cells represent approximately 35% of the lymphoid cells in the liver, 25% in lungs and 15% in the spleen but only 0.2% of the nucleated cells in the lymph nodes.^36^ This highlights the importance of delivering nanovaccines not only to a compartment with an increased number of iNKT cells but also with increased numbers of relevant cellular partners to obtain more efficient antigen-specific T and B cell responses.

Even though iNKT cells and iNKT1 subset are less prevalent in the lymph nodes, lymph node resident iNKT17 cells could be activated via either subcutaneous or intranodal injections of nanoparticles. Upon activation, these iNKT cells were also capable of maturating DCs and activating T cells within the lymph nodes (Figure 5). Throughout the study, probably depending on the splenic drainage of each peripheral injection, we observed some occasional IFN- γ secretion and highly variable cytotoxic T cell responses after subcutaneous and intranodal injections. However, the abundant numbers of T, B and NK cells in the spleen were mostly exempted from stimulation after these injections as evidenced by the lower proliferation, CD107a, PD-1, and CD69 upregulation on OT-I T cells, lower antibody levels and CD69 upregulation on B and NK cells. We recently demonstrated that CCR4 upregulation on naïve T cells after systemic α-GalCer administration was dependent on both TCR and IL-4 R ligation in the spleen (Manuscript in print). We also observed a similar upregulation of CCR4 on OT-I T cells in spleen but no clear distinction was observed in the draining lymph nodes which require further investigation.

Drainage of nanoparticles to the liver was highest after iv injections. Nevertheless, we observed similar levels of activation on transferred OT-I T cells, total host CD8^+^ T cells, B cells, NK cells, and cDC1’s in the liver independent of the route of administration. This could be the result of very high numbers of iNKT cells in the liver which are capable of secreting high amounts of IFN-γ and provide help to OT-I T cell priming in the liver.^37-39^ This is in line with previous findings where, α-GalCer treatment was reported to recruit lymphocytes toward the liver including NK cells and NKT cells, further increasing their numbers.^3,37^ On the other hand, it is well known that lymphocytes are extensively recruited to the spleen after α-GalCer treatment and both the spleen architecture and its cellular components are more specialized to support T cell priming. Our data indicate higher levels of OT-I T cell activation in the spleen when compared to the liver despite a large number of nanoparticles draining to the liver. These findings emphasize the importance of splenic drainage to obtain robust T cell cytotoxicity and long term tumor control.

Comparing administration routes in a therapeutic tumor model also supported the advantages of intravenous injection. The contrast between routes was even more pronounced at lower doses of the nanovaccine possibly due to lesser splenic drainage as evidenced by the loss of systemic IFN-γ which is a key element in anti-tumor immunity. The high levels of activation obtained by iv injections came with a price of PD-1 upregulation on T cells. Affirmatively, PD-1 blockage combination only synergized with intravenous vaccination leading to extended survival. This is in line with the suggested prediction of the IFN-γ signature for clinical responses upon anti-PD-1 treatment.^40^ We observed that anti-PD-1 antibody treatment extends the cytotoxic capacity of T cells without affecting the circulating numbers in intravenously vaccinated naïve mice. For the anti-4-1BB agonistic antibody, we observed both increased IFN- γ levels and extended control of HPV antigen-expressing TC-1 tumors. This synergy between vaccination and anti-4-1BB antibody was reported previously in the same tumor model albeit via intranasal instillation of peptide and α-GalCer mixtures conferring higher cytotoxic potential to T cells but did not demonstrate any benefit from anti-PD-1 antibody treatment.^27^ Here, we observed synergistic effects of both combinatorial approaches in the same tumor model via intravenous administration of nanoparticles containing 10 fold less peptide and iNKT cell agonists. In summary, our results support the notion that proper activation of cytotoxic T cells is critical in tumor control, and a combination of iv nanoparticle vaccination and anti-PD-1 or anti-4-1BB antibody treatments leads to more durable tumor control.

In this study, we revealed that the anti-tumor efficacy of iNKT cell vaccines relies on the presence of subsets of iNKT cells in the vicinity of the APC. Given the specific distribution of these cells, access to iNKT cell rich tissues that are also inhabited by relevant cellular partners such as particular DC subpopulations, NK cells, and potentially other subsets is of critical importance for the optimal responses of nanovaccines that contain iNKT cell agonists. We demonstrated that, even though the subcutaneous and intranodal injections of nanoparticles drain efficiently to lymph nodes, they are impaired of accessing spleen and liver where critical NKT1 population and NK cells are present. The results unequivocally show that the intravenous route gives rise to the most potent antigen-specific Th1 type T and B cell responses and could be successfully combined with checkpoint modulation for the treatment of established tumors. We believe that this study provides thus far overlooked aspects of iNKT cell-mediated immunotherapy and provides new insights into how iNKT cell-activating cancer vaccines should be applied in the clinical trials.

## Supplementary Material

Supplemental Material

## Data Availability

The datasets used and/or analyzed during the current study are available from the corresponding author on reasonable request.

## List of abbreviations

iNKT cells: Invariant natural killer T cells
NK cells: natural killer cells
NP: Nanoparticle
IFN: Interferon
PD-1: Programmed cell death protein 1
MHC: Major histocompatibility complex
IL: Interleukin
α-GalCer: α-galactosylceramide
PLGA: poly(lactic-co-glycolic acid)
OVA: Ovalbumin protein
IMM60: threitolceramide 6
TCR: T cell receptor
CAD: Charged Aerosol Detector
Ig: Immunoglobulin
Iv: intravenous injection
Sc: subcutaneous injection
Inod: intranodal injection
Spl: Spleen
LN: Lymph node
Axil: Axillary
Ing: Inguinal
ALT: Alanine aminotransferase
