# Supplementary material for: Nanovaccine administration route is critical to obtain pertinent iNKt cell help for robust anti-tumor T and B cell responses
Source: Oncoimmunology. 2020 Mar 17;9(1):1738813. doi: 10.1080/2162402X.2020.1738813 (PMC7790498; doi:10.1080/2162402X.2020.1738813)
Supplement: Supplemental Material [file KONI_A_1738813_SM3348.zip › Supplementary Figure caption.docx]

**Supplementary Figure-1: Comparison of antigen distribution delivered by nanoparticles or soluble form.** Distribution of ovalbumin either encapsulated in PLGA nanoparticles or delivered in solution. Wild-type BALB/c mice were injected with 1mg of PLGA nanoparticles containing 41.7ug ovalbumin-vivotag750 (NP(OVA) orange), 0,74mg of PLGA nanoparticles containing 41.7µg ovalbumin-vivotag750 and 87.3 ng of IMM60 (NP(OVA+IMM60) purple) or 41.7ug ovalbumin-vivotag750 dissolved in PBS (sol OVA green) via intravenous (iv) route. **A)** Representative full-body images demonstrating the overall distribution of different formulations after 3 and 24 hours. **B)** Mice were euthanized at different time points, images of excised organs were acquired separately and fluorescence was quantified in each ROI (Larger ROI size was applied for all Livers, same ROI size was applied to all other organs). **C)** Alanine transaminase activity measured from 24h serum samples. Each mouse is depicted as a dot with mean values of groups N=2 per time point per formulation. (iv) red, (sc) green, (inod) blue

**Supplementary Figure 2:** A, B, C) In vitro cytokine secretion profiles of iNKT cells from different mice organs. Differing amounts of total cells isolated from wild type C57BL/6 mice were incubated with 1ug/mL IMM60 for 24 hours. IFN-γ (A), IL-4 (B), and IL-17A (C) levels in culture media were analyzed by ELISA. D) The percentage of all iNKT cells within all T cells were demonstrated from the same experiment shown in Figure-4A. Each mouse is depicted as a dot with mean values of groups Two-way ANOVA was used for statistical analysis.

**Supplementary Figure-3:** As in figure 5. Wild-type C57BL/6 mice were injected with 0,172 mg of PLGA nanoparticles (containing 6ug Ovalbumin and 30ng IMM60) via different routes. 24 hours later spleens and lymph nodes were isolated. **A)** Percentages of CD40+ cells among XCR-1^-^ CD11b^+^ cDC2s in spleen (left) and in lymph nodes (right). **B)** Percentages of CD40+ cells among XCR-1^+^ cDC1s in the spleen, injected and non injected inguinal lymph nodes after intranodal injections. **C)** Percentages of PD-1+ cells among transferred OT-I T cells in spleen (left) and ipsilateral lymph nodes (right). **D)** Percentages of CD107a+ cells among transferred OT-I T cells in spleen (left) and ipsilateral lymph nodes (right). **E)** Percentages of CCR4+ cells among transferred OT-I T cells in spleen (left) and ipsilateral lymph nodes (right).

**Supplementary Figure-4:** As in sup.figure-3 and figure-5. Wild-type C57BL/6 mice were injected with 0,172 mg of PLGA nanoparticles (containing 6ug Ovalbumin and 30ng IMM60) via different routes. 24 hours later spleens, lymph nodes, and livers were isolated. **A)** Percentages of CD69+ cells among host mice CD8+ T cells in spleen (left) and in lymph nodes (middle) and livers (right). **B)** Percentages of CD69+ cells among host mice CD19+ B cells in spleen (left) and in lymph nodes (middle) and livers (right). **C)** Percentages of CD69+ cells among host mice CD3^-^ NK1.1^+^ NK cells in spleen (left) and livers (right). **D)** Percentages of CD69+ cells among host mice CD3^+^ NK1.1^+^ NKT cells in spleen (left) and livers (right). **E)** Histograms demonstrating the expression levels of CD69 on different cell subsets in spleen after iv injection of NP(OVA) (blue) or NP(OVA+IMM60) (red). Kruskal-Wallis test followed by uncorrected Dunn’s test; One-way or Two-way ANOVA followed by Uncorrected Fisher's LSD was used for statistical analysis.

**Supplementary Figure-5: A-D)** As in figure 7A-C**,** Wild-type C57BL/6 mice were intravenously injected with 0,172 mg of PLGA nanoparticles (containing 6ug Ovalbumin and 30ng IMM60) with or without intraperitoneal PD-1 or PD-L1 injections repeated every 3 days. Mice were transferred with ovalbumin peptide-loaded target cells on days 6 and 13 after vaccinations **A**) KLRG1+ percentage and **B)** NK1.1+ percentage of iNKT cells in spleen as determined by fluorescent CD1d-α-GalCer Dextramer binding in different treatment groups on 7 and 14 days after vaccination. **C)** Percentage of NK cells among all viable cells in spleen as determined by CD1d-α-GalCer Dextramer negative, CD3 negative NK1.1 positive cells in different treatment groups on 7 and 14 days after vaccination. **D**) KLRG1 percentage of NK cells in spleen shown in C. **E)** Serum IFN-y levels of mice vaccinated with NP(HPV+IMM60) 24 hours after nanoparticle injection. **F)** Serum IFN-y levels of mice shown in figure 7D-E. Serum samples were collected 18 hours after 2nd vaccinations. Each dot represents a mouse.

**Supplementary Figure-6: A)** Demonstration of B16.ova tumor growth on each mouse as in Figure 6-E. **B)** The demonstration of TC-1 tumor growth on each mouse is shown as in Figure 7-D-F. * Tumor free mice at the end of the study.

**Supplementary Figure-7: A)** Gating strategy applied for DC subset and maturation analysis. Initially, single cells with low viability dye were morphologically gated to exclude granulocytes, autofluorescent red pulp macrophages were excluded based on a non-stained empty fluorescent channel and B cells were excluded based on CD19 expression. CD11c^hi^ CD11b^low^ cells with high MHC-II expression were selected as cDCs. XCR-1^+^ CD11b^-^ cells (cDC1) and XCR-1^-^ CD11b^low^ cells (cDC2) were further analyzed by CD40 and MHC-II expression levels. **B)** Demonstrative difference between groups in antigen-specific cytotoxicity on days 7 and 14 shown in Figure-7A.
