# Supplementary figures and images for: Nanovaccine administration route is critical to obtain pertinent iNKt cell help for robust anti-tumor T and B cell responses
Source: Oncoimmunology. 2020 Mar 17;9(1):1738813. doi: 10.1080/2162402X.2020.1738813 (PMC7790498; doi:10.1080/2162402X.2020.1738813)

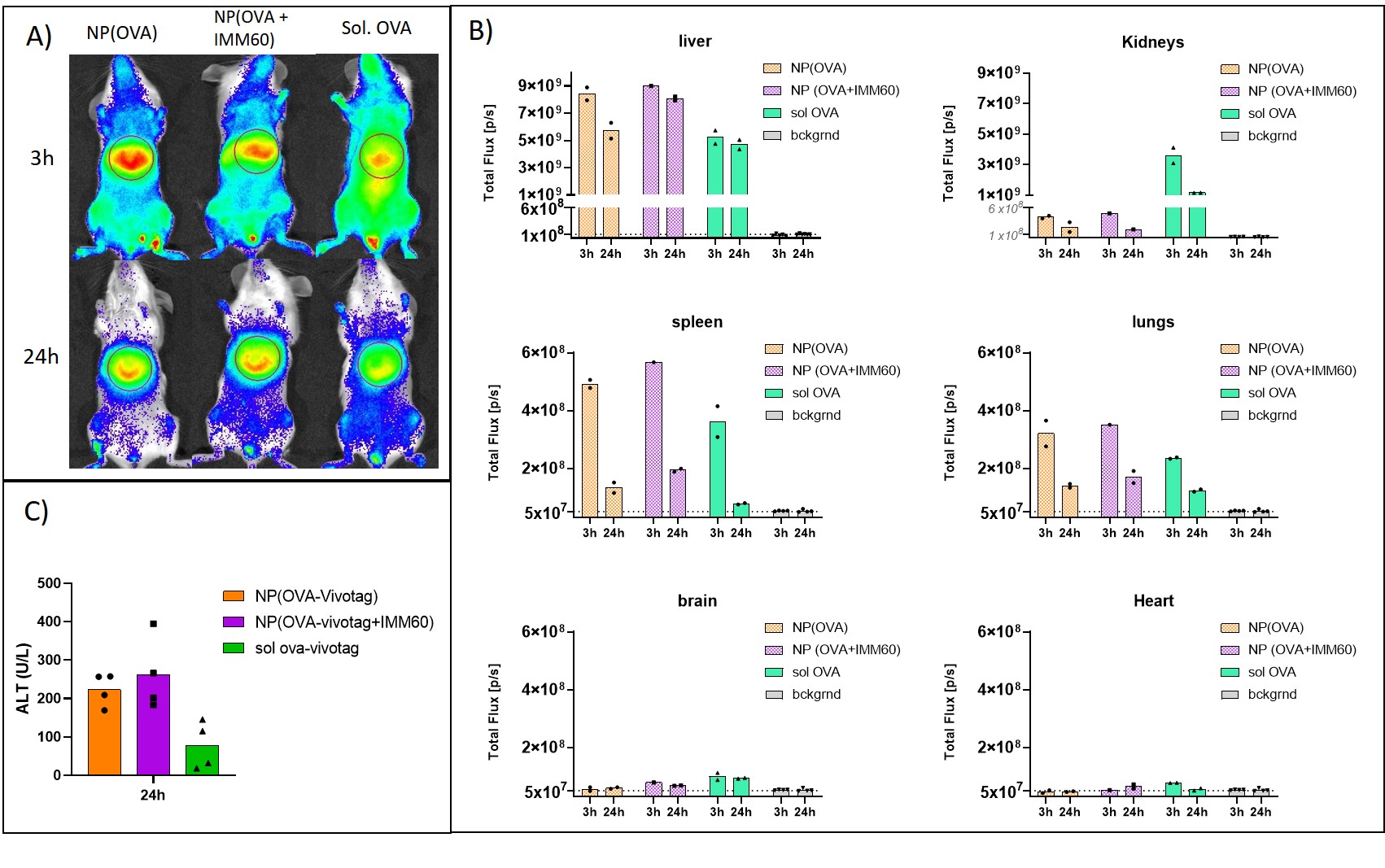

Supplement: Supplemental Material [file KONI_A_1738813_SM3348.zip › sup-1.jpg]

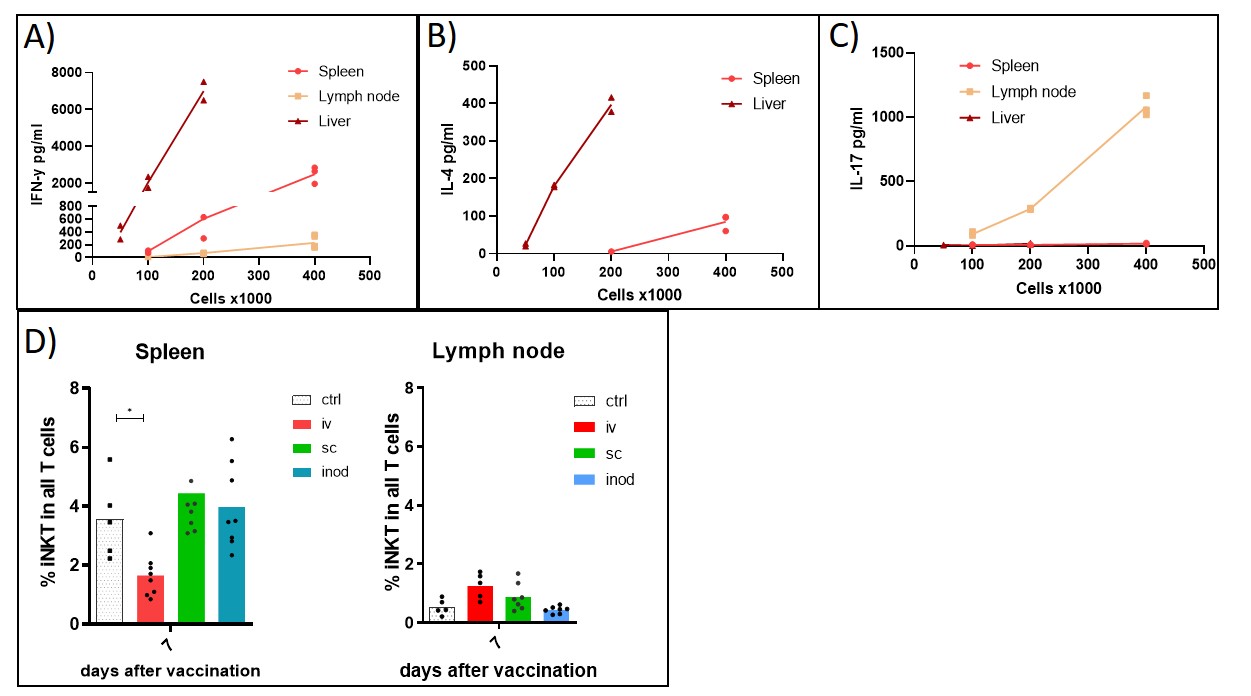

Supplement: Supplemental Material [file KONI_A_1738813_SM3348.zip › sup-2.jpg]

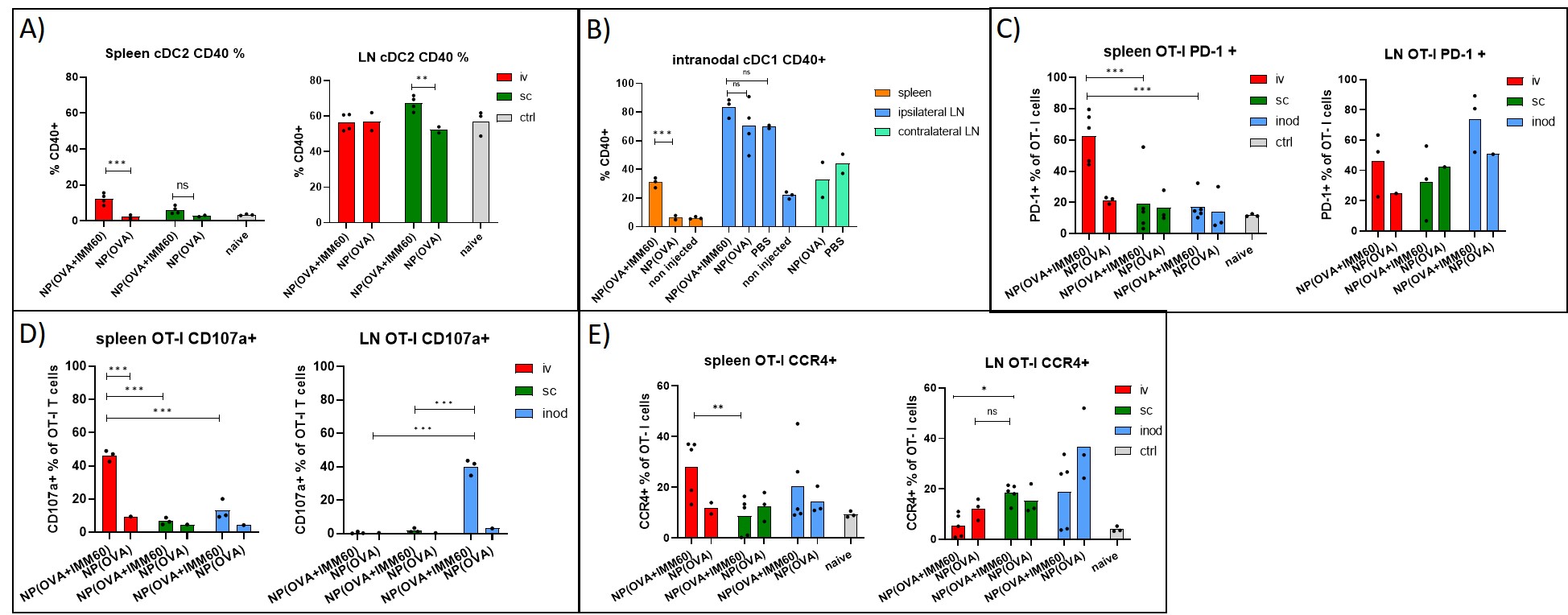

Supplement: Supplemental Material [file KONI_A_1738813_SM3348.zip › sup-3.jpg]

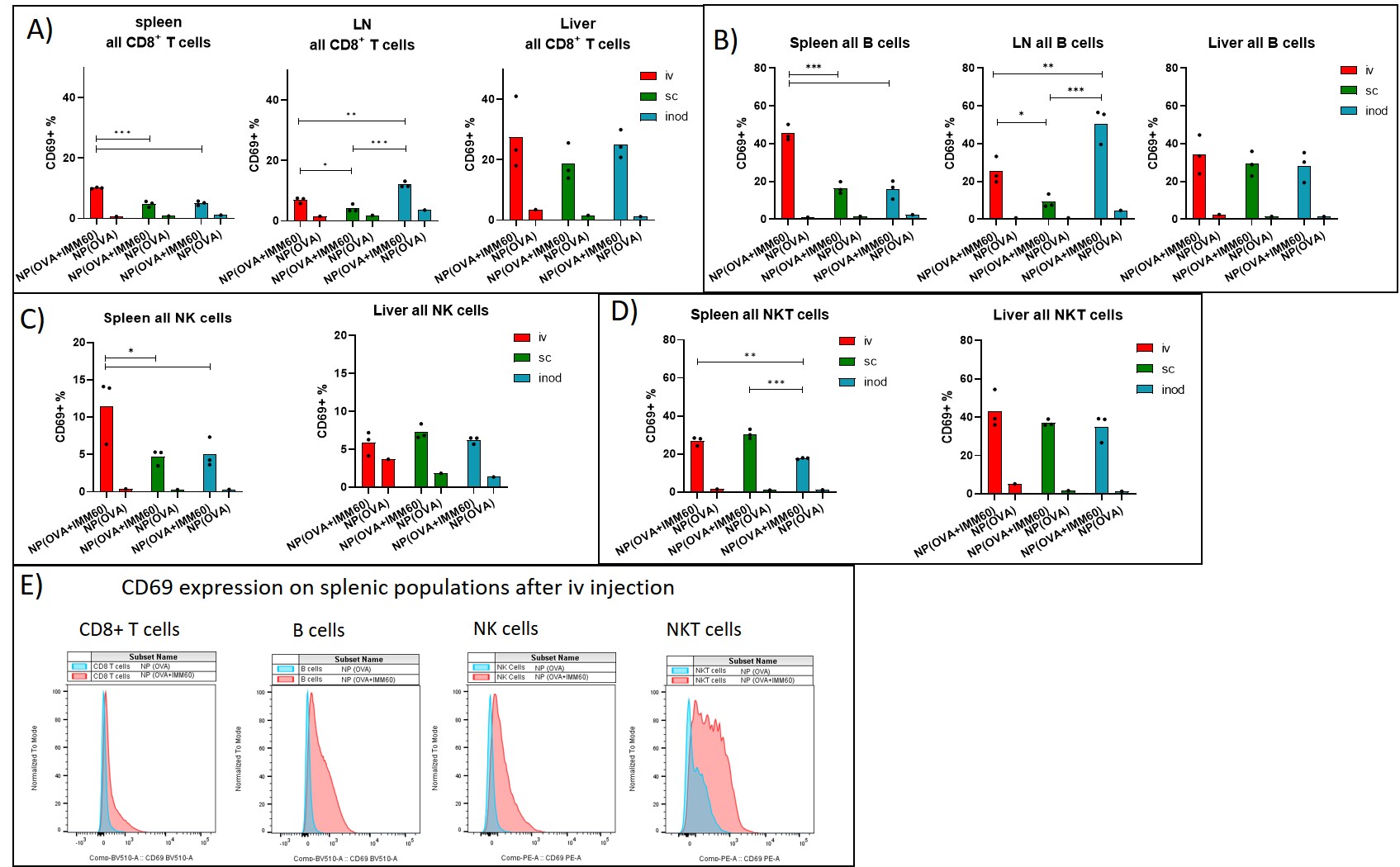

Supplement: Supplemental Material [file KONI_A_1738813_SM3348.zip › sup-4.jpg]

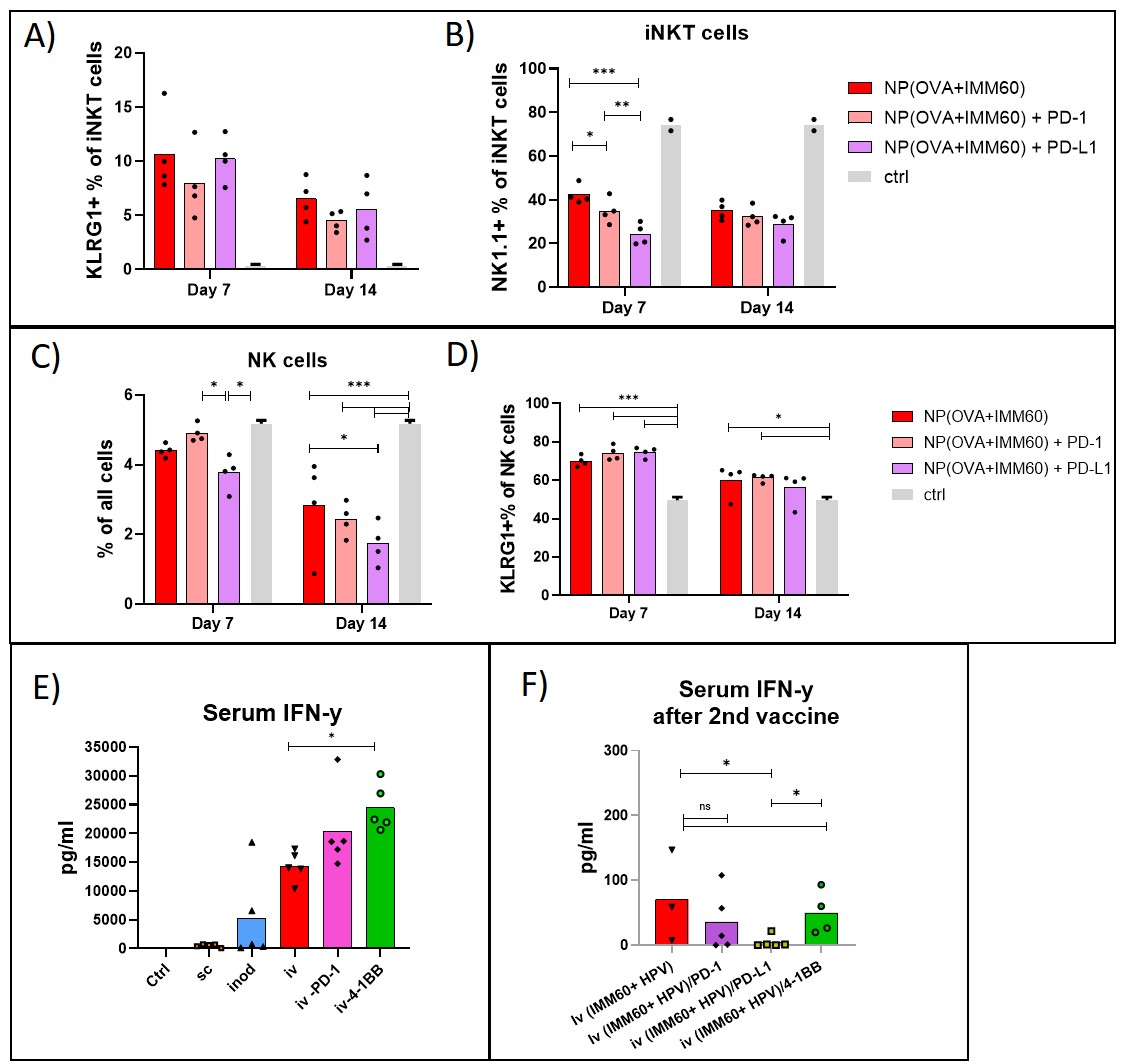

Supplement: Supplemental Material [file KONI_A_1738813_SM3348.zip › sup-5.jpg]

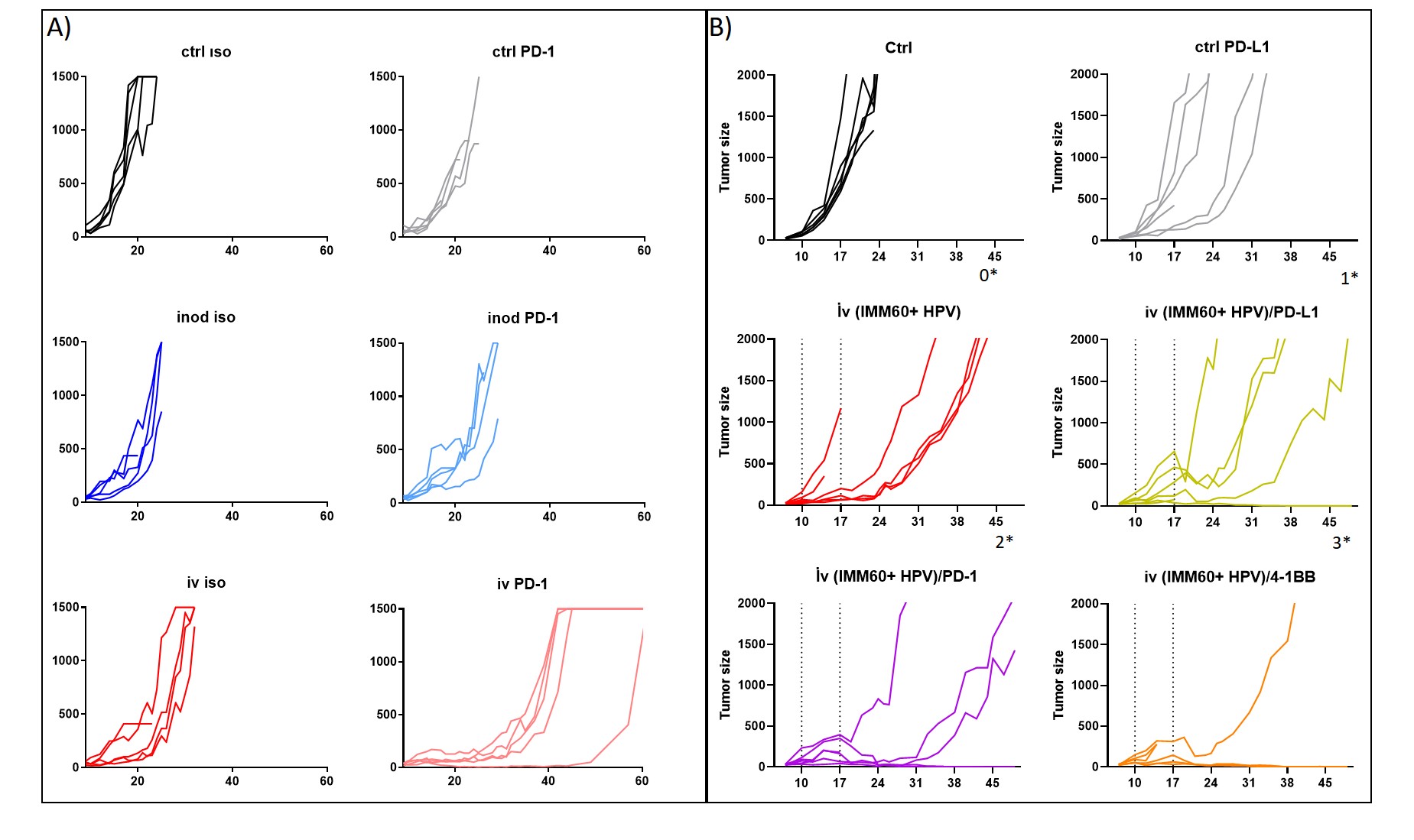

Supplement: Supplemental Material [file KONI_A_1738813_SM3348.zip › sup-6.jpg]

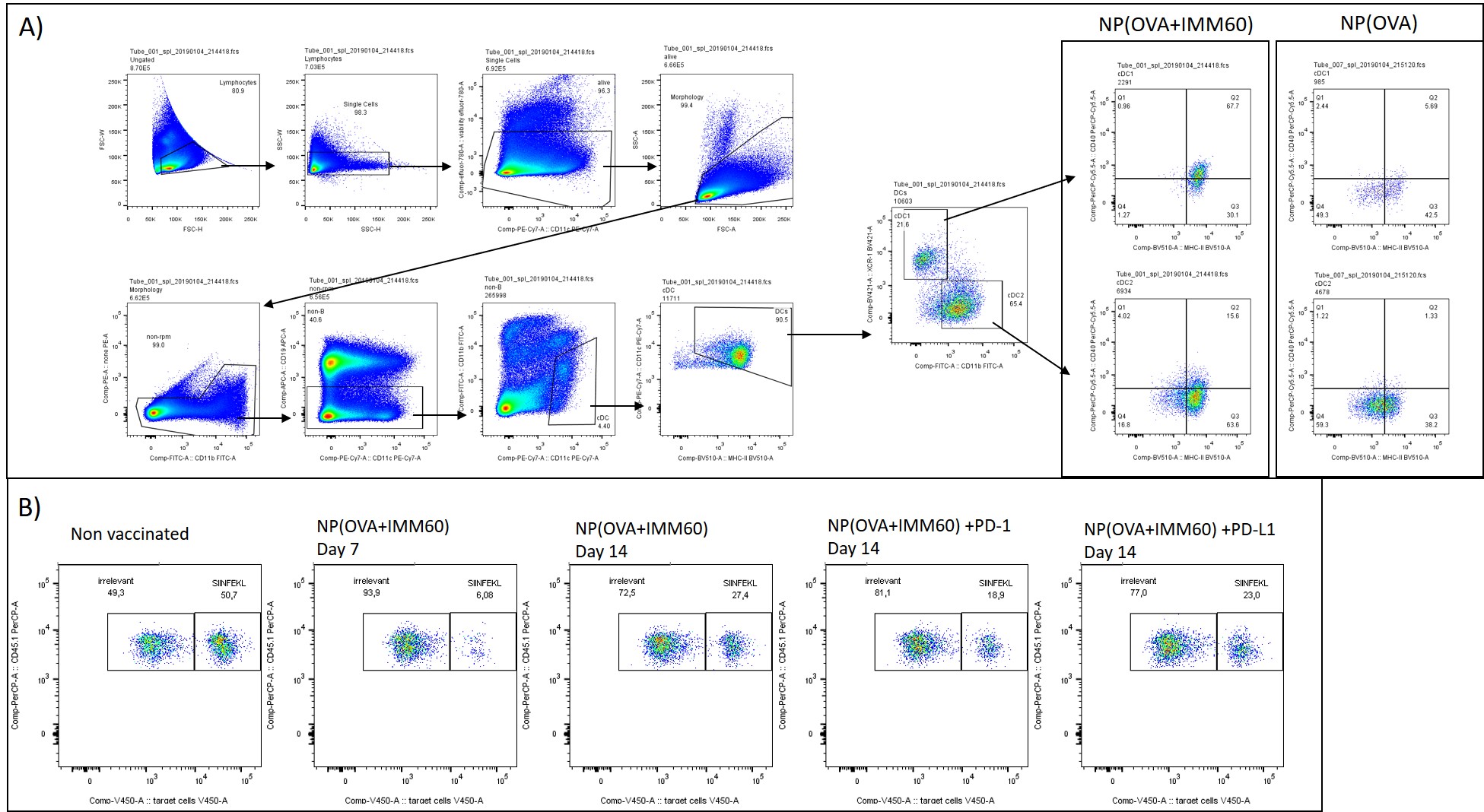

Supplement: Supplemental Material [file KONI_A_1738813_SM3348.zip › sup-7.jpg]
